# Synthesis and Biological Evaluation of Thalidomide Derivatives as Potential Anti-Psoriasis Agents

**DOI:** 10.3390/ijms19103061

**Published:** 2018-10-07

**Authors:** Kai-Wei Tang, Zih-Chan Lin, Yeh-Long Chen, Cherng-Chyi Tzeng, Jia-You Fang, Chih-Hua Tseng

**Affiliations:** 1 School of Pharmacy, College of Pharmacy, Kaohsiung Medical University, Kaohsiung 807, Taiwan; dadaking1107@gmail.com; 2 Graduate Institute of BioMedical Sciences, Chang Gung University, Taoyuan 333, Taiwan; sevenaurora@gmail.com; 3 Department of Medicinal and Applied Chemistry, College of Life Science, Kaohsiung Medical University, Kaohsiung 807, Taiwan; yeloch@kmu.edu.tw (Y.-L.C.); tzengch@kmu.edu.tw (C.-C.T.); 4 Chinese Herbal Medicine Research Team, Healthy Aging Research Center, Chang Gung University, Taoyuan 333, Taiwan; 5 Research Center for Food & Cosmetic Safety & Research Center for Chinese Herbal Medicine, Chang Gung University of Science & Technology, Taoyuan 333, Taiwan; 6 Department of Anesthesiology, Chang Gung Memorial Hospital, Taoyuan 333, Taiwan; 7 Department of Fragrance & Cosmetic Science, College of Pharmacy, Kaohsiung Medical University, Kaohsiung 807, Taiwan; 8 Department of Medical Research, Kaohsiung Medical University-Hospital, Kaohsiung 807, Taiwan; 9 Department of Pharmacy, Kaohsiung Municipal Ta-Tung Hospital, Kaohsiung 807, Taiwan

**Keywords:** thalidomide, psoriasis, tumor necrosis factor, anti-inflammatory, anti-proliferative

## Abstract

Several thalidomide derivatives were synthesized and evaluated for their anti-inflammatory activity. Introduction of the benzyl group to the parent thalidomide is unfavorable in which 2-(1-benzyl-2,6-dioxopiperidin-3-yl)isoindoline-1,3-dione (**4a**) was inactivated. However, the inhibitory activities on TNF-α and IL-6 expression in HaCaT cells were improved by the substitution of a chloro- or methoxy- group at the phenyl position of **4a**. The IL-6 inhibitory activity decreased in an order of **5c** (69.44%) > **4c** (48.73%) > **6c** (3.19%) indicating the 3-substituted derivative is more active than the 4-substituted counterpart, which in turn is more active than the 2-substituted counterpart. Among them, 2-[1-(3-chlorobenzyl)-2,6-dioxopiperidin-3-yl]isoindoline-1,3-dione (**5c**) was found to inhibit TNF-α and IL-6 expression in HaCaT cells with a higher potency than thalidomide and no significant cell cytotoxicity was detected at 10 μM. In psoriasis, Compound **5c** reduced IL-6, IL-8, IL-1β and IL-24 in imiquimod-stimulated models. Our results indicated that compound **5c** is a potential lead of novel anti-psoriasis agents. Structural optimization of compound **5c** and its in vivo assay are ongoing.

## 1. Introduction

Psoriasis is a chronic inflammatory skin disorder that affects 2% of the world population [1]. The symptoms of psoriasis on the skin, such as pain, scaling, and itching, results in decreased quality of life and significant healthcare-related costs [2]. Moreover, patients suffering from moderate-to-severe plaque psoriasis may also suffer from increased risk of depression and cardiovascular disease [3]. The conventional treatments for moderate-to-severe plaque psoriasis, such as methotrexate, cyclosporine, retinoids, hydroxycarbamide, and fumarates, raise awareness of potential significant side effects [4,5]. A new approach to treat psoriasis is to down-regulate the specific cytokines and chemokines. Recently, certain biological treatments targeting specific immune pathways, such as tumor necrosis factor (TNF) (infliximab, adalimumab), T cells (alefacept), and the p40 subunit of interleukin (IL)-12/IL-23 (ustekinumab) were developed with good therapeutic effects [6,7,8]. However, the biologics still present some challenges, such as limited administration route, quality control, and high therapeutic costs. Moreover, some severe side effects have been reported, including reactivation of hepatitis B infection and increased risk of infection for HIV patients, after anti-TNF therapy [9]. Consequently, there is still an urgent need for the discovery of novel compounds that could improve the safety profile with good efficacy.

Thalidomide, a synthetic glutamic acid derivative, was initially developed for epilepsy treatment in the early 1950s. Following a lack of sufficient evidence of anticonvulsant efficacy, it was then marketed as a sedative medication and was also widely used for its antiemetic effect during pregnancy but was soon after withdrawn from the market because of its catastrophic teratogenicity [10,11]. In 1998, thalidomide was approved again for the treatment of erythema nodosum leprosum (ENL) [12,13]. Moreover, it has been rediscovered and marketed as having immunomodulatory and anti-angiogenetic properties [14]. TNF-α, an inflammatory cytokine involved in the pathogenesis of several inflammatory diseases including psoriasis, could be inhibited by thalidomide [15] and its derivatives such as 2-(1-benzyl-2,6-dioxopiperidin-3-yl)isoindoline-1,3-dione (**4a**) [16]. Thalidomide has also been found to inhibit basic fibroblast growth factor (bFGF), vascular endothelial growth factor (VEGF), interleukin-6 (IL-6) production, and tumor necrosis factor-induced nuclear factor-κB activation in Jurkat cells [14,17,18,19]. These cytokines, which result in suppression of inflammation, angiogenesis, and the immune response, play a crucial role in psoriasis [20,21,22]. Although it possesses multiple pharmacological applications and is widely used in clinical settings, it still possesses several induced side effects such as sedation, rash, constipation, peripheral neuropathy, dizziness, thromboembolism, and severe teratogenicity [23]. These results have raised great interest not only for clinical use of thalidomide but also the discovery of novel derivatives for better activities and decreased side effects.

**Figure 1:**
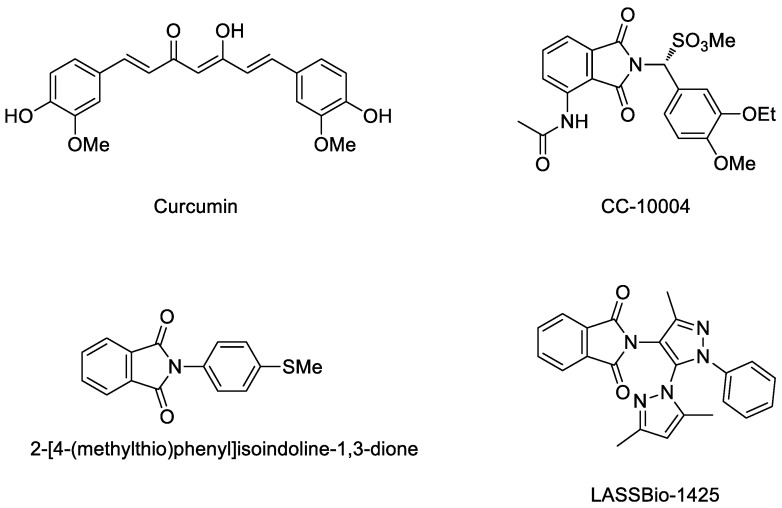
The chemical structures of curcumin, CC-10004, 2-[4-(methyl-thio)phenyl] isoindoline-1,3-dione, and LASSBio-1425.

Recently, several new inhibitors (Figure 1) have been in development for such skin diseases. Curcumin has been reported to be an anti-psoriasis drug candidate by interrupting certain cytokines including TNF-α, IL-17, IL6 and INF-γ [24]. CC-10004 was synthesized and developed as an oral treatment for psoriasis [25]. (2-[4-(Methyl-thio)phenyl]isoindoline-1,3-dione) and LASSBio-1425 have been reported as promising anti-inflammatory agents by interrupting TNF-α [26,27]. Strong evidence given by these studies is that the aryl- group and phthalimide are needed for anti-inflammatory activity in psoriasis.

We have also synthesized certain polycyclic heterocycles such as furo[3,2:3,4] naphtha[1,2-*d*]imidazole, benzo[*f*]indole-4,9-dione and indeno[1,2-*c*]quinoline derivatives for the evaluation of their anti-inflammatory activities [28,29,30,31,32]. Herein, we describe the discovery of a novel series of thalidomide derivatives that connect thalidomide and selected substituted aryl- groups by different linkers. Our design is aimed at retaining the anti-TNF-α and anti-ILs pharmacological profiles from thalidomide and to enhance the efficacy against specific cytokines related to psoriasis.

## 2. Results and Discussion

### 2.1. Chemistry

**Scheme 1:**
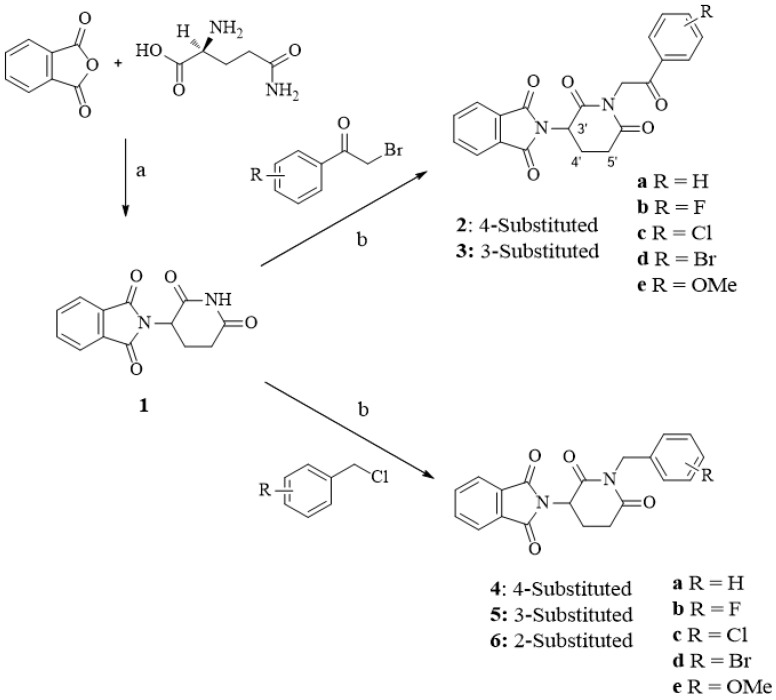
Synthesis of thalidomide derivatives **2a**–**6e**.

The synthesis of compounds **2a**–**6e** is depicted in Scheme 1. Compound **1** (thalidomide) was first prepared from phthalic anhydride and (l)-glutamine under the condition of acetic anhydride, Et_3_N and toluene according to the research published by Rao, D.R., et al. 2009 [33]. 2-(1-Benzyl-2,6-dioxopiperidin-3-yl)isoindoline-1,3-dione (**4a**) was reported in the research of Luzzio, F.A., et al. 2003 and synthesized by two-step using phthalimidoglutaric anhydride and benzyl amine as starting material [34]. However, the drawbacks of these synthetic strategies are low-yield, high-cost, and complexity of method. In the present study, the new classes of thalidomide analogues were prepared by the modification of the glutarimide ring using one-step synthetic strategy to add selected benzyl and phenacyl groups. Synthesis of compounds **2a**–**6e** was accomplished by the substitution of thalidomide (**1**) with selected benzyl chloride and phenacyl bromide under basic conditions of potassium carbonate. The structures of final compounds were confirmed by ^1^H NMR, ^13^C NMR spectra (spectra data can be found in Supplementary Materials) and elemental analysis as described in Section 4. 

### 2.2. Biology

#### 2.2.1. In Vitro Anti-Psoriasis Activity

##### Thalidomide Derivatives Attenuated TNF-α and IL-6 Production

Production of inflammatory cytokines, including TNF-α, IL-1β, IL-6, IL-8 and IL-24 in the skin is increased in various chronic skin diseases including atopic dermatitis and psoriasis [35]. Recently, topical application of imiquimod (IMQ) on mouse skin exacerbated psoriasis at both the local treated areas as well as distant sites, which has led to the development of pre-clinical models of psoriasis using topically applied IMQ cream [36]. Moreover, Schön et al. reported that imiquimod induced upregulation of proinflammatory cytokines including TNF-α, IL-1β, IL-6, and IL-8 in Toll-like receptor (TLR)7- and TLR8-negative cells, such as the keratinocyte-derived cell line HaCaT [37]. Therefore, all the synthesized thalidomide derivatives were evaluated for inhibition of imiquimod (IMQ)-induced TNF-α and IL-6 production in HaCaT cells, and cell viability. The preliminary results of these thalidomide derivatives are summarized in Table 1. Thalidomide (**1**), which inhibits IL-6 (48.70%) and TNF-α (22.97%) expression in HaCaT cells at 5 μM, was used as a positive control. 2-[2,6-Dioxo-1-(2-oxo-2-phenylethyl)piperidin-3-yl]isoindoline-1,3-dione (**2a**) was inactivated indicating the introduction of 2-oxo-ethylphenyl side chain in the parent thalidomide is unfavorable. Substitution of bromo atom at the 4-phenyl position of **2a** improved IL-6 and TNF-α inhibitory activities in which **2d** was weakly active against IL-6 (14.86%). However, its inhibitory activity on TNF-α (30.38%) expression was more potent than thalidomide (22.97%). Compounds **3b**, **3c**, and **3e** were more active than **2b**, **2c**, and **2e** respectively against IL-6 expression, indicating 3-substituted derivatives are superior to their 4-substituted counterparts. Introduction of the benzyl group to the parent thalidomide was also unfavorable in which compound **4a** became inactive. However, substitution at 4-phenyl position, such as compounds **4b**–**e**, improved IL-6 inhibitory activity. Among them, compound **4c** exhibited comparable IL-6 inhibitory activity to thalidomide (**1**). The IL-6 inhibitory activity decreased in an order of **5c** (69.44%) > **4c** (48.73%) > **6c** (3.19%) indicating 3-substituted derivative was more active than the 4-substituted counterpart, which in turn was more active than the 2-substituted counterpart. The same trend was observed in which the IL-6 inhibitory activity decreased in an order of **5e** (62.12%) > **4e** (14.43%) > **6e** (2.09%). For 3-substituted derivatives, 3-fluoro derivative **5b** was inactive while 3-chloro derivative **5c** and 3-methoxy derivative **5e** possessed about 1.4-fold stronger IL-6 inhibitory activity than thalidomide. In TNF-α inhibitory activity evaluation, compounds **2d**, **2e**, **5c**, and **6b** exhibited more active inhibitory effect than thalidomide. Among them, 2-[1-(3-chlorobenzyl)-2,6-dioxopiperidin-3-yl]isoindoline-1,3-dione (**5c**) possessed the most potent inhibition of TNF-α, being about 3.4-fold more active than thalidomide and was non-cytotoxic at the concentration of 10 µM. Our results indicated that **5c** exhibited more potent inhibitory activity than thalidomide to both IL-6 and TNF-α simultaneously.

##### Effect of Compound **5c** on Cell Viability

**Figure 2:**
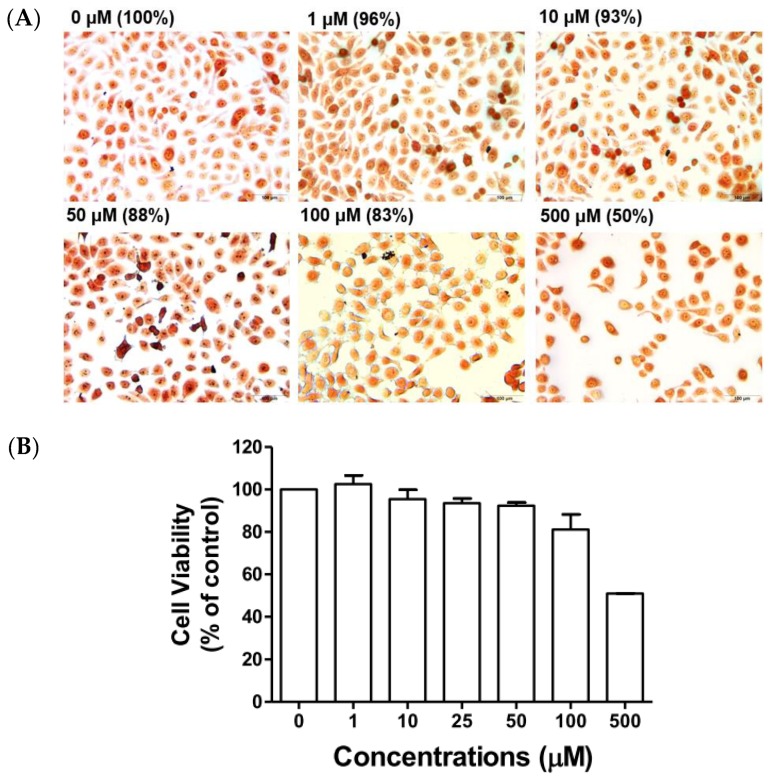
HaCaT cells were treated with 1–500 μM of **5c** respectively and cell viability was assessed by Neutral Red assay. Data are presented as mean ± SD (*n* = 3).

To determine the cytotoxic effect of **5c** on HaCaT cells, cell viability was assessed by performing neutral red staining assay. Neutral red staining is widely used for measuring the cell viability or cytotoxicity. The morphological characteristics of the HaCaT cells were illustrated using a phase-contrast microscope. The viable HaCaT cells incorporate neutral red in lysosomes whereas, dead or damaged cells do not take up the dye (Figure 2A). HaCaT cells were treated with various concentrations of **5c** ranging from 1 to 500 μM. As shown in Figure 2B, compound **5c** showed over 80% of cell viability in 100 μM and over 90% of cell viability in 50 μM, which indicated that **5c** possessed excellent inhibitory activity of proinflammatory cytokines with low cytotoxicity.

##### Compound **5c** Attenuated IL-1β, IL-6, IL-8 and IL-24 Production

**Figure 3:**
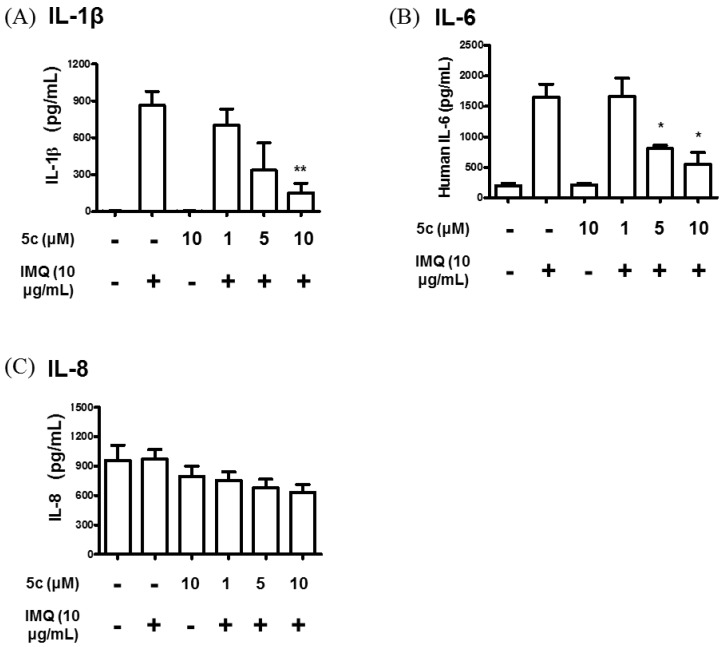
Effect of **5c** on inhibition of inflammatory cytokines in differentiated HaCaT cells was done by ELISA (**A**) IL-1β, (**B**) IL-6 and (**C**) IL-8. The cells were incubated with IMQ alone, IMQ with different concentrations of **5c** in media containing 2% fetal bovine serum (FBS) for 24 h at 37 °C. Data are presented as mean ± SD (*n* = 3).

**Figure 4:**
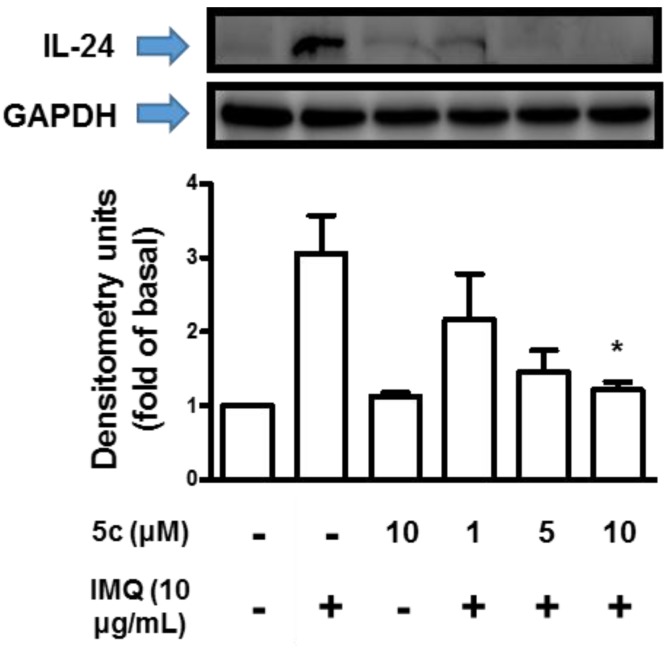
Effect of **5c** on inhibition of IL-24 in differentiated HaCaT cells was done by Western Blot. The cells were incubated with IMQ alone, IMQ with different concentrations of **5c** in media containing 2% FBS for 24 h at 37 °C. Data are presented as mean ± SD (*n* = 3).

To evaluate the effect of interleukin family related to psoriasis treated with compound **5c**, HaCaT cells was stimulated with IMQ (10 μg/mL) and assessed by ELISA or western blot. As shown in Figure 3A, the amount of IL-1β decreased over 80% in the presence of 10 μM compound **5c** and the dose-dependent effect was revealed in IMQ-stimulated assay. In Figure 3B, IL-6 secreted by cells stimulated with IMQ showed apparent decrease with 5 and 10 μM compounds **5c**. The dose-dependent effect was observed by the IL-6 secretion of the cells treated with IMQ. The results of IL-8 are shown in Figure 3C. Compound **5c** caused limited effect on IL-8 in IMQ-treated cells but the attenuation of IL-8 and the slightly dose-dependent effect could still be observed. In Figure 4, secretion of IL-24 was almost inhibited with 10 μM compound **5c** in IMQ-stimulated western blot assay and the dose-dependent effect could be observed in IMQ-stimulated assay.

##### Compound **5c** Inhibited MAPKs Signaling Transduction Pathway 

**Figure 5:**
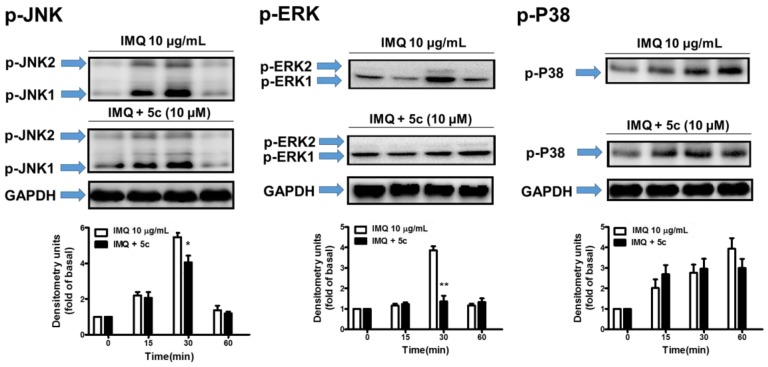
Effect of **5c** on inhibition of p-JNK1/2, p-ERK1/2 and p-38 in differentiated HaCaT cells was done by Western Blot. The cells were incubated with IMQ alone, IMQ with different concentrations of **5c** in media containing 2% FBS for 24 h at 37 °C. Data are presented as mean ± SD (*n* = 3).

**Figure 6:**
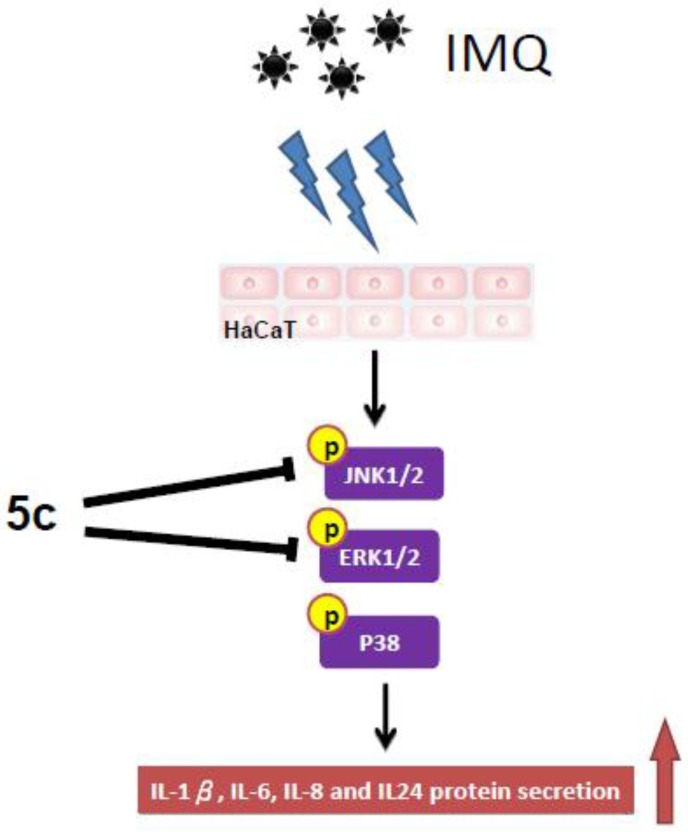
The supposition of mitogen-activated protein kinases (MAPKs) regulated proinflammatory pathways in psoriasis.

Mitogen-activated protein kinases (MAPKs), regulated key proinflammatory pathways following stimulation with environmental stimuli. MAPKs signaling pathways, including ERK, p38, and JNK, can regulate cellular responses to proliferation, apoptosis, differentiation, and inflammation in humans. Previous studies also found that MAPKs and NF-κB pathways play an important role in IMQ-induced psoriasiform dermatitis [38,39]. To confirm the actual pharmacological mechanism of compound **5c** on psoriasis, p-JNK1/2, p-ERK1/2 and p-P38 were assessed by western blot using HaCaT cells treated with IMQ and compound **5c,** with cells treated by IMQ alone used as control. The results are shown in Figure 5. Compared to the control, the test group incubated with compound **5c** revealed significant decrease in p-JNK1/2 and p-ERK1/2 assay over 30 to 60 min; however, there was no inhibitory effect on p-P38 assay. The results provide strong evidence that the decrease of IL-1β, IL-6, IL-8 and IL-24 affected by compound **5c** might act on MAPKs pathways in psoriasis via the disruption of JNK1/2 and ERK1/2, but not P38 (Figure 6). 

#### 2.2.2. Molecular Docking Study on TNF-α, IL-6, JNK1, ERK2

**Figure 7:**
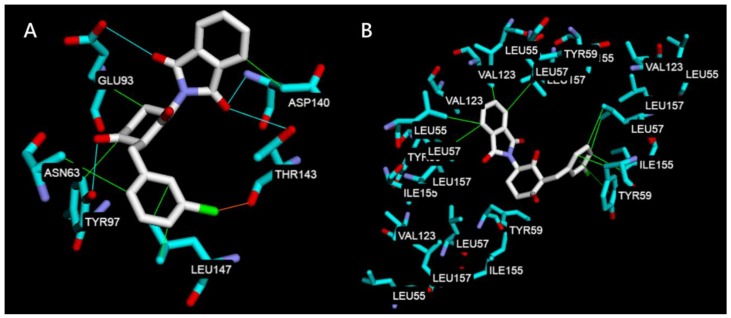
Docking poses of compound **5c** at the (**A**) IL-6 (PDB code 1ALU) and (**B**) TNF-α (PDB code 2AZ5). The interaction illustrated by green lines represents hydrophobic interaction, blue lines represent hydrogen bonding, and orange line represents halogen bonding.

To rationalize the obtained biological results, a molecular docking study was used to evaluate the potential interaction mechanism between compound **5c** and TNF-α, IL-6, JNK1, ERK2. Compound **5c** was first docked at the IL-6 (PDB code 1ALU) [40] and TNF-α (PDB code 2AZ5) [41] subunit interface. The docking poses of compound **5c** were examined and the pose that had the lowest binding energy is shown in Figure 7. In Figure 7A, compound **5c** performs three kinds of interaction with IL-6 including hydrophobic interaction with Leu147, Asp140, Glu93, Asn63 and Tyr97, hydrogen bonds with Glu93, Asp140, Thr143 and Tyr97, and halogen bond with Thr143. However, the lowest binding energy score of IL-6 performed only −7.40 kcal/mol. The results indicate that the decrease of IL-1β, IL-6, IL-8, and IL-24 in biological analyses may involve in other mechanism but not act directly by compound **5c**. In Figure 7B, compound **5c** only interacts through hydrophobic interaction by the aromatic rings and the lowest binding energy score of TNF-α was −9.80 kcal/mol. The results could be explained in that compound **5c** was able to dock into the middle of TNF-α dimer to form a stable complex and indicate that it may be the one of mechanisms of anti-inflammatory activity. The molecular docking study shown in Figure 7 shared the same results of the pharmacological test where inflammation of psoriasis may be inhibited by compound **5c** through the MAPKinase pathway via the inhibitory effect(s) of ERK1/2 and JNK1/2.

**Figure 8:**
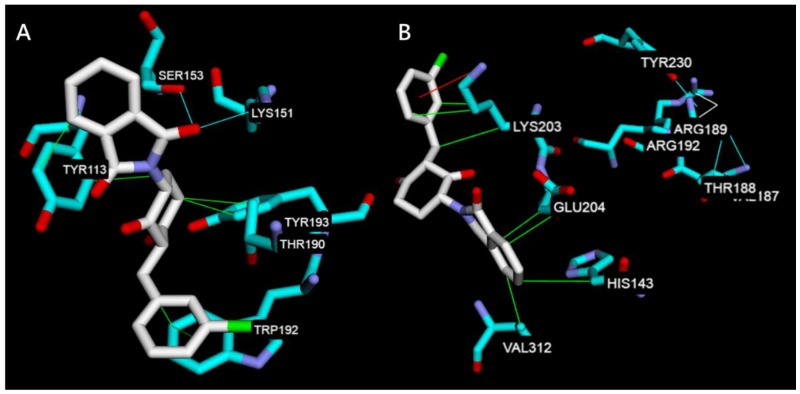
Docking poses of compound **5c** at the (**A**) ERK2 (PDB code 2Y9Q) and (**B**) JNK-1 (PDB code 3PZE). The interaction illustrated by green lines represents hydrophobic interaction, blue lines represent hydrogen bonding and red line represents pi-cation.

Figure 8A illustrates the docking results of compound **5c** and ERK2 (PDB code 2Y9Q) [41] where hydrophobic interactions were formed between the aromatic rings and Tyr113, Trp 192 and hydrogen bonds were built to Ser153 and Lys151. Figure 8B shows the docking results of compounds **5c** and JNK1 (PDB code 3PZE) [42] where hydrophobic interactions were also formed through the aromatic rings to Lys203, Glu204, His143, Val312 and pi-cation interaction was stacked to Lys203. The lowest binding energy score of ERK2 (−9.10 kcal/mol) was lower than JNK1 (−7.80 kcal/mol), indicating the same results of the western blot assays of p-ERK1/2 and p-JNK1/2.

## 3. Conclusions

A series of novel thalidomide derivatives were synthesized and evaluated for anti-inflammatory activity. Among them, 2-[1-(3-chlorobenzyl)-2,6-dioxopiperidin-3-yl] isoindoline-1,3-dione (**5c**) was found to inhibit TNF-α and IL-6 expression in HaCaT cells with a higher potency than thalidomide and no significant cell cytotoxicity was detected at 10 µM. We also determined some structural activity relationships between the thalidomide derivatives and inhibitory effects of IL-6 and TNF-α. Anti-psoriasis mechanism studies have indicated that compound **5c** reduced the secretion of IL-1β, IL-6, IL-8 and IL-24 in an IMQ-stimulated model and could also attenuate the activation of IMQ-induced MAPKs, including JNK1/2 and ERK1/2. These results suggest that compound **5c** successfully amplified both anti-inflammatory and immunomodulatory activities. Compound **5c** could be used as a candidate immunomodulatory agent in inflammatory skin disease, especially in psoriasis; however, its use in the case of TNF-α-overexpressing skin disease should be further investigated.

## 4. Materials and Methods

### 4.1. Chemistry Section

General Information. Commercial reagents were used as received without additional purification. Melting points were determined with Electrothermal IA9100 micro-melting point apparatus and uncorrected. NMR spectra were recorded with Varian Unity-400MHz spectrometer using DMSO-*d*_6_ as solvent and tetramethylsilane as internal standard. Chemical shifts were expressed as δ (ppm). Splitting patterns have been described as follows: s = singlet; d = doublet; t = triplet; q = quartet; dd = double doublet; m = multiplet. Analytical TLC was performed on Art. 5554 Kieselgel 60 GF254 produced by E. Merck and the spots of compounds were detected with UV light indicator irradiated at 254 and 366 nm. Art. 7734 Kieselgel 60 GF254 (70–400 mesh) made by E. Merck was used for column chromatography. The purity of compounds was determined with HPLC and elemental analysis (EA). HPLC analysis was performed on HITACHI Chromaster 5110 HPLC, fitted with a UV detector and an auto sampler. The compounds were tested on a Mightysil RP-18 GP 250-4.6 (5 mm) with mobile phase 10 mM KH_2_PO_4_:acetonitrile (35:65) pH = 2.89. The flow rate was 1 mL/min and the sample injection was 100 µL (5.00 mg dissolved in 1 mL DMSO, then diluted with methanol to 5 mL). The wavelength was set at 295 nm. Elemental Analysis was recorded on Heraeus CHN-O Rapid apparatus and the results were within ±0.4% of the theoretical value. 

#### 4.1.1. General Procedure for the Synthesis of Compounds **2a**–**3c**

The mixture of the thalidomide (**1**, 0.52 g, 2.00 mmol), potassium carbonate (1.38 g, 10.0 mmol) and acetone (20.0 mL) was stirred at room temperature for 10 min. Selected phenacyl bromide (2.40 mmol) was then added into the mixture and stirred at room temperature for 24 h (monitored by TLC). The mixtures were concentrated under reduced pressure first and extracted twice by dichloromethane/water (1/1) 80 mL. The organic layers were dried with magnesium sulfate and concentrated under reduced pressure. The samples were further purified with silica gel column chromatography, using dichloromethane as the mobile phase, to produce compounds with variable yields (35–63%).

*2-[2,6-Dioxo-1-(2-oxo-2-phenylethyl)piperidin-3-yl]isoindoline-1,3-dione* (**2a**). Compound **2a** was obtained in 48% yield as a light-yellow solid. Melting Point: 148.5–150.6 °C. Purity 98.0%. ^1^H NMR (400 MHz, DMSO-*d*_6_): δ 8.06–8.05 (m, 2H, Ar–H), 7.96–7.89 (m, 4H, Ar–H), 7.72–7.69. (m, 1H, Ar–H), 7.59–7.55 (m, 2H, Ar–H), 5.36 (dd, 1H, *J* = 13.2, 5.2 Hz, 3’-H), 5.24, 5.17 (AB system, 2H, *J* = 28 Hz, NCH_2_), 3.25–2.22 (4m, 4H, 4’-H and 5’-H).^13^C NMR (100 MHz, DMSO-*d*_6_): δ 191.91 (C=O), 171.30, 169.25 , 167.06 (C_1_, C_3_, C_2__’_, C_6’_), 134.91 (2C), 134.27, 133.98, 131.19 (2C), 128.92 (2C), 127.97 (2C), 123.44 (2C), 49.61 (NCH_2_), 46.41 (C_3’_), 30.98 (C_5’_), 21.11 (C_4’_). Anal. calcd. for C_21_H_16_N_2_O_5_: C, 67.02; H, 4.28; N, 7.44. Found: C, 67.42; H, 4.44; N, 7.30.

*2-{1-[2-(4-Fluorophenyl)-2-oxoethyl]-2,6-dioxopiperidin-3-yl} isoindoline-1,3-dione* (**2b**). Compound **2b** was obtained in 48% yield as a light-yellow solid. Melting Point: 139.7–141.2 °C. Purity 98.9%. ^1^H NMR (400 MHz, DMSO-*d*_6_) δ 8.17–8.12 (m, 2H, Ar–H), 7.96–7.88 (m, 4H, Ar–H), 7.43–7.38 (m, 2H, Ar–H), 5.38 (dd, 1H, *J* = 5.2, 13.2 Hz, 3’-H), 5.24, 5.17 (AB system, 2H, *J* = 26.8 Hz, NCH_2_), 3.18–2.19 (4m, 4H, 4’-H and 5’-H). ^13^C NMR (100 MHz, DMSO-*d*_6_) δ 190.63 (C=O), 171.30, 169.25, 167.06 (C_1_, C_3_, C_2__’_, C_6’_), 165.41 (^1^*J*_CF_ = 251.7 Hz), 134.91 (2C), 131.18 (2C), 131.11 (2C, ^3^*J*_CF_ = 9.9 Hz), 123.43 (2C), 116.00 (2C, ^2^*J*_CF_ = 22.0 Hz), 49.60 (NCH_2_), 46.34 (C_3’_), 30.95 (C_5’_), 21.10 (C_4’_). Anal. calcd. for C_21_H_15_FN_2_O_5_: C, 63.96; H, 3.83; N, 7.10. Found: C, 63.74; H, 3.93; N, 7.01.

*2-{1-[2-(4-Chlorophenyl)-2-oxoethyl]-2,6-dioxopiperidin-3-yl}isoindoline-1,3-dione* (**2c**). Compound **2c** was obtained in 49% yield as a light-yellow solid. Melting Point: 147.2–149.6 °C. Purity 96.4%. ^1^H NMR (400 MHz, DMSO-*d*_6_) δ 8.08–8.05 (m, 2H, Ar–H), 7.95–7.89 (m, 4H, Ar–H), 7.66–7.63 (m, 2H, Ar–H), 5.36 (dd, 1H, *J* = 5.2, 13.2 Hz, 3’-H), 5.23, 5.16 (AB system, 2H, *J* = 28.4 Hz, NCH_2_), 3.17–2.20 (4m, 4H, 4’-H and 5’-H). ^13^C NMR (100 MHz, DMSO-*d*_6_) δ 191.18 (C=O), 171.30, 169.25, 167.06 (C_1_, C_3_, C_2__’_, C_6’_), 138.92, 134.92 (2C), 132.95, 131.18 (2C), 129.94 (2C), 129.06 (2C), 123.44 (2C), 49.61 (NCH_2_), 46.38 (C_3’_), 30.96 (C_5’_), 21.10 (C_4’_). Anal. calcd. for C_21_H_15_ClN_2_O_5_: C, 61.40; H, 3.68; N, 6.82. Found: C, 61.23; H, 3.80; N, 6.70.

*2-{1-[2-(4-Bromophenyl)-2-oxoethyl]-2,6-dioxopiperidin-3-yl}isoindoline-1,3-dione* (**2d**). Compound **2d** was obtained in 63% yield as a light-yellow solid. Melting Point: 115.1–116.9 °C. Purity 97.8%. ^1^H NMR (400 MHz, DMSO-*d*_6_) δ 8.00–7.97 (m, 2H, Ar–H), 7.96–7.89 (m, 4H, Ar–H), 7.80–7.77 (m, 2H, Ar–H), 5.37 (dd, 1H, *J* = 5.2, 13.2 Hz, 3’-H), 5.22, 5.15 (AB system, 2H, *J* = 28.8 Hz, NCH_2_), 3.22–2.16 (4m, 4H, 4’-H and 5’-H). ^13^C NMR (100 MHz, DMSO-*d*_6_) δ 191.40 (C=O), 171.30, 169.25, 167.06 (C_1_, C_3_, C_2__’_, C_6’_), 134.93 (2C), 133.27, 132.01 (2C), 131.19 (2C), 130.02 (2C), 128.15, 123.45 (2C), 49.59 (NCH_2_), 46.35 (C_3’_), 30.96 (C_5’_), 21.10 (C_4’_). Anal. calcd. for C_21_H_15_BrN_2_O_5_: 0.25 H_2_O: C, 54.85; H, 3.40; N, 6.09. Found: C, 54.63; H, 3.27; N, 5.86.

*2-{1-[2-(4-Methoxyphenyl)-2-oxoethyl]-2,6-dioxopiperidin-3-yl}isoindoline-1,3-dione* (**2e**). Compound **2e** was obtained in 49% yield as a yellow solid. Melting Point: 179.9–181.4 °C. Purity 96.2%. ^1^H NMR (400 MHz, DMSO-*d*_6_) δ 8.05–8.02 (m, 2H, Ar–H), 7.96–7.89 (m, 4H, Ar–H), 7.10–7.07 (m, 2H, Ar–H), 5.36 (dd, 1H, *J* = 5.2, 13.2 Hz, 3’-H), 5.18, 5.06 (AB system, 2H, *J* = 27.8 Hz, NCH_2_), 3.86 (s, 3H, O–CH_3_), 3.18–2.17 (4m, 4H, 4’-H and 5’-H). ^13^C NMR (100 MHz, DMSO-*d*_6_) δ 190.05 (C=O), 171.30, 169.22, 167.07 (C_1_, C_3_, C_2__’_, C_6’_), 163.64, 134.91 (2C), 131.19 (2C), 130.34 (2C), 127.21, 123.42 (2C), 114.12 (2C), 55.59 (OCH_3_), 49.64 (NCH_2_), 46.09 (C_3’_), 31.00 (C_5’_), 21.12 (C_4’_). Anal. calcd. for C_22_H_18_N_2_O_6_: C, 65.02; H, 4.46; N, 6.89. Found: C, 65.04; H, 4.49; N, 6.82.

*2-{1-[2-(3-Fluorophenyl)-2-oxoethyl]-2,6-dioxopiperidin-3-yl}isoindoline-1,3-dione* (**3b**). Compound **3b** was obtained in 25% yield as a yellow solid. Melting Point: 164.0–165.9 °C. Purity 95.9%. ^1^H NMR (400 MHz, DMSO-*d*_6_) δ 7.97–7.89 (m, 4H, Ar–H), 7.88–7.85 (m, 2H, Ar–H), 7.65–7.58 (m, 2H, Ar–H), 5.39 (dd, 1H, *J* = 5.2, 13.2 Hz, 3’-H), 5.25, 5.18 (AB system, 2H, *J* = 24 Hz, NCH_2_), 3.22–2.17 (4m, 4H, 4’-H and 5’-H). ^13^C NMR (100 MHz, DMSO-*d*_6_) δ 191.21 (C=O), 171.35, 169.29, 167.10 (C_1_, C_3_, C_2__’_, C_6’_), 162.22 (^1^*J*_CF_ = 244.1 Hz), 136.34 (^3^*J*_CF_ = 6.8 Hz), 134.97 (2C), 131.28, 131.21 (2C), 124.31 (^3^*J*_CF_ = 2.3 Hz), 123.48 (2C), 121.00 (^2^*J*_CF_ = 21.2 Hz), 114.66 (^2^*J*_CF_ = 22 Hz), 49.60 (NCH_2_), 46.60 (C_3’_), 31.00 (C_5’_), 21.12 (C_4’_). Anal. calcd. for C_21_H_15_FN_2_O_5_: 0.1 H_2_O: C, 63.66; H, 3.87; N, 7.07. Found: C, 63.69; H, 3.82; N, 6.69.

*2-{1-[2-(3-Chlorophenyl)-2-oxoethyl]-2,6-dioxopiperidin-3-yl}isoindoline-1,3-dione* (**3c**). Compound **3c** was obtained in 34% yield as a light-yellow solid. Melting Point: 123.7–125.0 °C. Purity 97.6%. ^1^H NMR (400 MHz, DMSO-*d*_6_) δ 8.08–8.01 (m, 2H, Ar–H), 7.96–7.89 (m, 4H, Ar–H), 7.79–7.77 (m, 1H, Ar–H), 7.61 (t, *J* = 8 Hz, 1H, Ar–H), 5.39 (dd, 1H, *J* = 5.2, 13.2 Hz, 3’-H), 5.26, 5.19 (AB system, 2H, *J* = 28.4 Hz, NCH_2_), 3.22–2.17 (4m, 4H, 4’-H and 5’-H). ^13^C NMR (100 MHz, DMSO-*d*_6_) δ 191.29 (C=O), 171.32, 169.27, 167.08 (C_1_, C_3_, C_2__’_, C_6’_), 136.05, 134.95 (2C), 133.91, 133.74, 131.19 (2C), 130.95, 127.70, 126.74, 123.46 (2C), 49.59 (NCH_2_), 46.50 (C_3’_), 30.96 (C_5’_), 21.11 (C_4’_). Anal. calcd. for C_21_H_15_ClN_2_O_5_ **^.^** 0.1H_2_O: C, 61.11; H, 3.72; N, 6.78. Found: C, 60.86; H, 3.98; N, 6.89.

*2-{1-[2-(3-Methoxyphenyl)-2-oxoethyl]-2,6-dioxopiperidin-3-yl}isoindoline-1,3-dione* (**3e**). Compound **3e** was obtained in 63% yield as a light-yellow solid. Melting Point: 139.7–140.3 °C. Purity 96.9%. ^1^H NMR (400 MHz, DMSO-*d*_6_) δ 7.96–7.89 (m, 4H, Ar–H), 7.67–7.65 (m, 1H, Ar–H), 7.53–7.47 (m, 2H, Ar–H), 7.29–7.26 (m, 1H, Ar–H), 5.37 (dd, 1H, *J* = 5.2, 13.2 Hz, 3’-H), 5.23-5.17 (AB system, 2H, 24 Hz, NCH_2_), 3.83 (s, 3H, O-CH_3_), 3.22–2.17 (4m, 4H, 4’-H and 5’-H). ^13^C NMR (100 MHz, DMSO-*d*_6_) δ 191.74 (C=O), 171.32, 169.26, 167.09 (C_1_, C_3_, C_2__’_, C_6’_), 159.52, 135.62, 134.92 (2C), 131.20 (2C), 130.14, 123.45 (2C), 120.44, 120.13, 112.42, 55.39 (OCH_3_), 49.64 (NCH_2_), 46.60 (C_3’_), 31.00 (C_5’_), 21.13 (C_4’_). Anal. calcd. for C_22_H_18_N_2_O_6_: C, 65.02; H, 4.46; N, 6.89. Found: C, 65.00; H, 4.43; N, 6.87.

#### 4.1.2. General Procedure for the Synthesis of Compounds **4a**–**6e**

The mixture of the thalidomide (**1**, 0.52 g, 2.00 mmol), potassium carbonate (1.38 g, 10.0 mmol) and acetone (20.0 mL) was stirred in room temperature for 10 min. Selected benzyl chloride (2.40 mmol) was then added into the mixture and stirred in room temperature for 4 days (monitored by TLC). The mixtures were concentrated under reduced pressure first and extracted twice by dichloromethane/water (1/1) 80 mL. The organic layers were dried with magnesium sulfate and concentrated under reduced pressure. The samples were further purified with silica gel column chromatography, using dichloromethane as the mobile phase, to produce compounds with variable yields (30–70%).

*2-(1-Benzyl-2,6-dioxopiperidin-3-yl)isoindoline-1,3-dione* (**4a**). Compound **4a** was obtained in 30% yield as a light-yellow solid. Melting Point: 172.2–173.5 °C. Purity 98.2%. ^1^H NMR (400 MHz, DMSO-*d*_6_) δ 7.97–7.88 (m, 4H, Ar–H), 7.33–7.22 (m, 5H, Ar–H), 5.38 (dd, 1H, *J* = 5.2, 13.2 Hz, 3’-H), 4.93, 4.80 (AB system, 2H, *J* = 49.8 Hz, NCH_2_), 3.09-2.12 (4m, 4H, 4’-H and 5’-H). ^13^C NMR (100 MHz, DMSO-*d*_6_) δ 171.55, 169.56, 167.11 (C_1_, C_3_, C_2__’_, C_6’_), 136.96, 134.89 (2C), 131.19 (2C), 128.21 (2C), 127.05 (2C), 126.90, 123.45 (2C), 49.59 (NCH_2_), 42.74 (C_3’_), 31.09 (C_5’_), 21.15 (C_4’_). Anal. calcd. for C_20_H_16_N_2_O_4_: C, 68.96; H, 4.63; N, 8.04. Found: C, 68.74; H, 4.71; N, 7.86.

*2-[1-(4-Fluorobenzyl)-2,6-dioxopiperidin-3-yl]isoindoline-1,3-dione* (**4b**). Compound **4b** was obtained in 48% yield as a light-yellow solid. Melting Point: 193.9–194.2 °C. Purity 98.8%. ^1^H NMR (400 MHz, DMSO-*d*_6_) δ 7.97–7.88 (m, 4H, Ar–H), 7.33–7.28 (m, 2H, Ar–H), 7.17–7.11 (m, 2H, Ar–H), 5.37 (dd, 1H, *J* = 5.2, 13.2 Hz, 3’-H), 4.89, 4.78 (AB system, 2H, *J* = 43.2 Hz, NCH_2_), 3.12–2.09 (4m, 4H, 4’-H and 5’-H). ^13^C NMR (100 MHz, DMSO-*d*_6_) δ 171.59, 169.58, 167.12 (C_1_, C_3_, C_2__’_, C_6’_), 162.22 (^1^*J*_CF_ = 241.1 Hz), 134.91 (2C), 133.18 (^3^*J*_CF_ = 3.1 Hz), 131.19 (2C), 129.38 (2C, ^2^*J*_CF_ = 7.6 Hz), 123.46 (2C), 114.97 (2C, ^2^*J*_CF_ = 20.5 Hz), 49.56 (NCH_2_), 42.11 (C_3’_), 31.09 (C_5’_), 21.15 (C_4’_). Anal. calcd. for C_20_H_15_FN_2_O_4_: C, 65.57; H, 4.13; N, 7.65. Found: C, 65.56; H, 4.09; N, 7.58.

*2-[1-(4-Chlorobenzyl)-2,6-dioxopiperidin-3-yl]isoindoline-1,3-dione* (**4c**). Compound **4c** was obtained in 32% yield as a white solid. Melting Point: 176.2–176.7 °C. Purity 98.0%. ^1^H NMR (400 MHz, DMSO-*d*_6_) δ 7.96–7.89 (m, 4H, Ar–H), 7.39–7.36 (m, 2H, Ar–H), 7.30–7.28 (m, 2H, Ar–H), 5.37 (dd, 1H, *J* = 5.2, 13 Hz, 3’-H), 4.89, 4.80 (AB system, 2H, *J* = 37.2 Hz, NCH_2_), 3.12-2.10 (4m, 4H, 4’-H and 5’-H). ^13^C NMR (100 MHz, DMSO-*d*_6_) δ 171.65, 169.63, 167.15 (C_1_, C_3_, C_2__’_, C_6’_), 136.04, 134.97 (2C), 131.65, 131.22 (2C), 129.19 (2C), 128.23 (2C), 123.51 (2C), 49.59 (NCH_2_), 42.24 (C_3’_), 31.12 (C_5’_), 21.19 (C_4’_). Anal. calcd. for C_20_H_15_ClN_2_O_4_: C, 62.75; H, 3.95; N, 7.32. Found: C, 62.50; H, 3.87; N, 7.27.

*2-[1-(4-Bromobenzyl)-2,6-dioxopiperidin-3-yl]isoindoline-1,3-dione* (**4d**). Compound **4d** was obtained in 46% yield as a light-yellow solid. Melting Point: 154.2–156.0 °C. Purity 97.5%. ^1^H NMR (400 MHz, DMSO-*d*_6_) δ 7.96–7.89 (m, 4H, Ar–H), 7.52–7.49 (m, 2H, Ar–H), 7.24–7.22 (m, 2H, Ar–H), 5.38 (dd, 1H, *J* = 5.2, 13.2 Hz, 3’-H), 4.87, 4.78 (AB system, 2H, *J* = 37.2 Hz, NCH_2_), 3.12-2.10 (4m, 4H, 4’-H and 5’-H). ^13^C NMR (100 MHz, DMSO-*d*_6_) δ 171.64, 169.61, 167.13 (C_1_, C_3_, C_2__’_, C_6’_), 136.46, 134.95 (2C), 131.21 (2C), 131.13 (2C), 129.54 (2C), 123.50 (2C), 120.10, 49.54 (NCH_2_), 42.25 (C_3’_), 31.10 (C_5’_), 21.16 (C_4’_). Anal. calcd. for C_20_H_15_BrN_2_O_4_ **^.^** 0.1H_2_O: C, 55.98; H, 3.58; N, 6.53. Found: C, 55.62; H, 3.58; N, 6.50.

*2-[1-(4-Methoxybenzyl)-2,6-dioxopiperidin-3-yl]isoindoline-1,3-dione* (**4e**). Compound **4e** was obtained in 40% yield as a white solid. Melting Point: 150.6–151.5 °C. Purity 98.6%. ^1^H NMR (400 MHz, DMSO-*d*_6_) δ 7.97–7.89 (m, 4H, Ar–H), 7.21–7.18 (m, 2H, Ar–H), 6.90–6.81 (m, 2H, Ar–H), 5.36 (dd, 1H, *J* = 5.2, 13.2 Hz, 3’-H), 4.85, 4.72 (AB system, 2H, *J* = 52.4 Hz, NCH_2_), 3.73 (s, 3H, O-CH_3_), 3.11-2.08 (4m, 4H, 4’-H and 5’-H). ^13^C NMR (100 MHz, DMSO-*d*_6_) δ 171.54, 169.53, 167.15 (C_1_, C_3_, C_2__’_, C_6’_), 158.32, 134.92 (2C), 131.22 (2C), 129.02, 128.87(2C), 123.47 (2C), 113.63 (2C), 55.04 (OCH_3_), 49.64 (NCH_2_), 42.24 (C_3’_), 31.15 (C_5’_), 21.16 (C_4’_). Anal. calcd. for C_21_H_18_N_2_O_5_: C, 66.66; H, 4.79; N, 7.40. Found: C, 66.99; H, 4.85; N, 7.33.

*2-[1-(3-Fluorobenzyl)-2,6-dioxopiperidin-3-yl]isoindoline-1,3-dione* (**5b**). Compound **17** was obtained in 55% yield as a white solid. Melting Point: 172.1–172.5 °C. Purity 96.0%. ^1^H NMR (400 MHz, DMSO-*d*_6_) δ 7.97–7.89 (m, 4H, Ar–H), 7.40–7.34 (m, 1H, Ar–H), 7.12–7.05 (m, 3H, Ar–H), 5.41 (dd, 1H, *J* = 5.2, 13.2 Hz, 3’-H), 4.94, 4.83 (AB system, 2H, *J* = 41.2 Hz, NCH_2_), 3.15-2.12 (4m, 4H, 4’-H and 5’-H). ^13^C NMR (100 MHz, DMSO-*d*_6_) δ 171.65, 169.68, 167.16 (C_1_, C_3_, C_2__’_, C_6’_), 162.21 (^1^*J*_CF_ = 241.9 Hz), 139.86 (^3^*J*_CF_ = 7.6 Hz), 134.96 (2C), 131.22 (2C), 130.21 (^2^*J*_CF_ = 8.3 Hz), 123.51 (2C), 123.08 (^4^*J*_CF_ = 2.2 Hz), 113.83 (^3^*J*_CF_ = 2.2 Hz), 113.62 (^2^*J*_CF_ = 3.8 Hz), 49.56 (NCH_2_), 42.35 (C_3’_), 31.09 (C_5’_), 21.21 (C_4’_). Anal. calcd. for C_20_H_15_FN_2_O_4_: C, 65.57; H, 4.13; N, 7.65. Found: C, 65.62; H, 4.04; N, 7.60.

*2-[1-(3-Chlorobenzyl)-2,6-dioxopiperidin-3-yl]isoindoline-1,3-dione* (**5c**). Compound **5c** was obtained in 51% yield as a white solid. Melting Point: 194.9–195.3 °C. Purity 96.1%. ^1^H NMR (400 MHz, DMSO-*d*_6_) δ 7.97–7.88 (m, 4H, Ar–H), 7.38–7.30 (m, 3H, Ar–H), 7.24–7.22 (m, 1H, Ar–H), 5.41 (dd, 1H, *J* = 5.2, 13.2 Hz, 3’-H), 4.92, 4.81 (AB system, 2H, *J* = 41.8 Hz, NCH_2_), 3.14-2.11 (4m, 4H, 4’-H and 5’-H). ^13^C NMR (100 MHz, DMSO-*d*_6_) δ 171.63, 169.65, 167.10 (C_1_, C_3_, C_2__’_, C_6’_), 139.46, 134.91 (2C), 132.97, 131.19 (2C), 130.10, 127.00, 126.97, 125.79, 123.46 (2C), 49.51 (NCH_2_), 42.31 (C_3’_), 31.06 (C_5’_), 21.16 (C_4’_). Anal. calcd. for C_20_H_15_ClN_2_O_4_: C, 62.75; H, 3.95; N, 7.32. Found: C, 62.99; H, 4.09; N, 7.04.

*2-[1-(3-methoxybenzyl)-2,6-dioxopiperidin-3-yl]isoindoline-1,3-dione* (**5e**). Compound **5e** was obtained in 70% yield as a white solid. Melting Point: 161.9–163.5 °C. Purity 96.4%. ^1^H NMR (400 MHz, DMSO-*d*_6_) δ 7.97–7.88 (m, 4H, Ar–H), 7.25–7.20 (m, 1H, Ar–H), 6.84–6.80 (m, 3H, Ar–H), 5.38 (dd, 1H, *J* = 5.2, 13.2 Hz, 3’-H), 4.91, 4.77 (AB system, 2H, *J* = 55.4 Hz, NCH_2_), 3.75 (s, 3H, OCH_3_), 3.14–2.11 (4m, 4H, 4’-H and 5’-H). ^13^C NMR (100 MHz, DMSO-*d*_6_) δ 171.54, 169.59, 167.13 (C_1_, C_3_, C_2__’_, C_6’_), 159.26, 138.53, 134.91 (2C), 131.20 (2C), 129.28, 123.45 (2C), 119.13, 112.55, 112.37, 54.92 (OCH_3_), 49.61 (NCH_2_), 42.70 (C_3’_), 31.09 (C_5’_), 21.17 (C_4’_). Anal. calcd. for C_21_H_18_N_2_O_5_: C, 66.66; H, 4.79; N, 7.40. Found: C, 66.68; H, 4.83; N, 7.07.

*2-[1-(2-Fluorobenzyl)-2,6-dioxopiperidin-3-yl]isoindoline-1,3-dione* (**6b**). Compound **6b** was obtained in 58% yield as a white solid. Melting Point: 160.2–161.2 °C. Purity 97.9%. ^1^H NMR (400 MHz, DMSO-*d*_6_) δ 7.96–7.87 (m, 4H, Ar–H), 7.33–7.27 (m, 1H, Ar–H), 7.23–7.14 (m, 3H, Ar–H), 5.39 (dd, 1H, *J* = 5.2, 13.2 Hz, 3’-H), 4.90 (s, 2H, NCH_2_), 3.15-2.12 (4m, 4H, 4’-H and 5’-H). ^13^C NMR (100 MHz, DMSO-*d*_6_) δ 171.58, 169.62, 167.15 (C_1_, C_3_, C_2__’_, C_6’_), 159.76 (^1^*J*_CF_ = 242.8 Hz), 134.97 (2C), 131.21 (2C), 128.91 (^3^*J*_CF_ = 8.1 Hz), 127.98 (^3^*J*_CF_ = 4.2 Hz), 124.35 (^4^*J*_CF_ = 3.1 Hz), 123.69 (^2^*J*_CF_ = 14.1 Hz), 123.52 (2C), 115.00 (^2^*J*_CF_ = 21.0 Hz), 49.61 (NCH_2_), 36.66 (C_3’_), 31.13 (C_5’_), 21.19 (C_4’_). Anal. calcd. for C_20_H_15_FN_2_O_4_: C, 65.57; H, 4.13; N, 7.65. Found: C, 65.55; H, 4.06; N, 7.62.

*2-[1-(2-Chlorobenzyl)-2,6-dioxopiperidin-3-yl]isoindoline-1,3-dione* (**6c**). Compound **6c** was obtained in 52% yield as a white solid. Melting Point: 166.1–167.0 °C. Purity 97.8%. ^1^H NMR (400 MHz, DMSO-*d*_6_) δ 7.96–7.88 (m, 4H, Ar–H), 7.47–7.45 (m, 1H, Ar–H), 7.35–7.28(m, 2H, Ar–H), 7.20–7.18 (m, 2H, Ar–H), 5.46 (dd, 1H, *J* = 5.2, 13.2 Hz, 3’-H), 4.93, 4.88 (AB system, 2H, *J* = 19.2 Hz, NCH_2_), 3.19–2.16 (4m, 4H, 4’-H and 5’-H). ^13^C NMR (100 MHz, DMSO-*d*_6_) δ 171.60, 169.63, 167.10 (C_1_, C_3_, C_2__’_, C_6’_), 134.94 (2C), 133.63, 131.44, 131.19 (2C), 129.14, 128.53, 127.22, 126.58, 123.48 (2C), 49.54 (NCH_2_), 40.70 (C_3’_), 31.09 (C_5’_), 21.19 (C_4’_). Anal. calcd. for C_20_H_15_ClN_2_O_4_: C, 62.75; H, 3.95; N, 7.32. Found: C, 62.30; H, 3.85; N, 7.23.

*2-[1-(2-Methoxybenzyl)-2,6-dioxopiperidin-3-yl]isoindoline-1,3-dione* (**6e**). Compound **6e** was obtained in 27% yield as a white solid. Melting Point: 202.2–204.0 °C. Purity 98.1%. ^1^H NMR (400 MHz, DMSO-*d*_6_) δ 7.96–7.89 (m, 4H, Ar–H), 7.25–7.21 (m, 1H, Ar–H), 7.00–6.97 (m, 2H, Ar–H), 6.92–6.88 (m, 1H, Ar–H), 5.43 (dd, 1H, *J* = 5.2, 13.2 Hz, 3’-H), 4.85, 4.77 (AB system, 2H, *J* = 30.4 Hz, N–CH_2_–Ar), 3.81 (s, 3H, OCH_3_), 3.18–2.15 (4m, 4H, 4’-H and 5’-H). ^13^C NMR (100 MHz, DMSO-*d*_6_) δ 171.57, 169.54, 167.13 (C_1_, C_3_, C_2__’_, C_6’_), 156.22, 134.91 (2C), 131.21 (2C), 127.78, 125.21, 124.07, 123.47 (2C), 120.10, 110.33, 55.33 (OCH_3_), 49.61 (NCH_2_), 38.25 (C_3’_), 31.14 (C_5’_), 21.22 (C_4’_). Anal. calcd. for C_21_H_18_N_2_O_5_ **^.^** 0.05H_2_O: C, 66.49; H, 4.82; N, 7.38. Found: C, 66.79; H, 4.88; N, 6.98.

### 4.2. Biological Activity

#### 4.2.1. Cell Culture

HaCaT cells (human keratinocytes cell) were a gift from Dr. Nan-Lin Wu at HsinChu Mackay Memorial Hospital. They were cultivated in DMEM complemented with 10% (*v*/*v*) FBS (fetal bovine serum), and penicillin/streptomycin mixture, in a water-jacketed CO_2_ incubator at 37 °C. Every 2 days, cells were passed using trypsin 0.05% (*w*/*v*).

#### 4.2.2. Cell Viability Assays

The cytotoxicity assays of the compounds toward HaCaT cells were conducted using MTT assay. Briefly, 5 × 10^4^ cells per well were seeded in a 96-well plate then incubated with different concentrations of the compounds at 37 °C for 24 h. Then, the cells were incubated with MTT solution (0.5 mg/mL) for 4 h at 37 °C. At the end of the incubation, the culture medium in each well was replaced by 150 μL of DMSO to dissolve the formazan-containing crystals. The plate was shaken for 5 min at room temperature, prior to measuring the absorbance of each well at 570 nm in a microplate reader. All the experiments were performed in triplicate.

#### 4.2.3. ELISA

Cells (1 × 10^5^/well) were seeded at least in triplicate into 12-well plates in 1ml medium with 10% FBS. After reaching confluence, the cells were washed and incubated with 0.5 ml serum-free medium containing the indicated concentration of imiquimod for 24 h. The culture supernatants were analyzed for IL-1β, IL-6 and IL-8 by ELISA (BioLegend, San Diego, CA, USA) according to the manufacturer’s instructions.

#### 4.2.4. Western Blotting Analysis

Equal proteins were separated on SDS-PAGE and transferred onto polyvinylidene difluoride (PVDF) membrane. Membranes were incubated with primary antibodies at 4 °C overnight, and then incubated with corresponding HRP-conjugated secondary antibodies for 1 h at room temperature. Protein bands were visualized using enhanced chemiluminescence reagent (NEN, Boston, MA, USA).

#### 4.2.5. Neutral Red Staining

Briefly, HaCaT cells (1 × 10^5^ cells/mL) were seeded into 6-well plates for overnight incubation at 37 °C with 5% CO_2_. Cells were treated with **5c** (0, 1, 10, 50, 100, 500 μM), then further incubated for 24 h at 37 °C, washed twice with 1× PBS post-incubation, fixed with 3.7% paraformaldehyde, and stained with neutral red dye for 60 min. The cells were again washed thrice with 1× PBS and visualized under the phase-contrast microscope.

### 4.3. Molecular Docking Study

The crystal structures of IL-6 (PDB ID: 1ALU), TNF-α (PDB ID: 2AZ5), ERK2 (PDB ID: 2Y9Q) and JNK1 (PDB ID: 3PZE) were downloaded from the RCSB Protein Date Bank. The 3D conformation of hit compound **5c** was generated by ChemBio 3D Ultra 14.0. The molecular docking was performed by Achilles Blind Docking Server (http://bio-hpc.ucam.edu/achilles/). The “blind docking” approach was used for the docking of the small molecule to the targets, which was done without *a priori* knowledge of the location of the binding site by the system [43]. Visual representation of molecules was created with 3Dmol by Nicholas Rego and David Koes [44].

## Figures and Tables

Reagent and conditions. (**a**) Acetic anhydride, triethylamine, toluene, reflux, 24 h; (**b**) potassium carbonate, acetone, stir rigorously, 1–4 days.

**Table 1:** Preliminary anti-psoriasis activity and cytotoxicities of thalidomide derivatives.

Compounds	% Inhibition at 5 µM		% Viability	
	IL-6	TNF-α	5 µM	10 µM
**Thalidomide (1)**	48.70 ± 1.10	22.97 ± 5.16	96.7 ± 3.4	86.7 ± 2.9
**2a**	3.85 ± 1.66	−2.45 ± 2.37	94.3 ± 2.5	86.0 ± 4.1
**2b**	3.33 ± 0.44	5.67 ± 0.13	96.3 ± 2.1	91.7 ± 3.9
**2c**	3.77 ± 0.56	12.93 ± 2.41	93.0 ± 4.3	94.7 ± 3.3
**2d**	14.86 ± 3.20	30.38 ± 8.49	90.7 ± 2.1	89.7 ± 6.2
**2e**	2.27 ± 1.66	39.77 ± 2.29	96.7 ± 2.1	84.0 ± 2.4
**3b**	17.51 ± 6.41	12.69 ± 6.50	96.2 ± 3.5	89.8 ± 3.8
**3c**	4.56 ± 2.91	8.28 ± 6.87	100.0 ± 0.3	98.1 ± 0.8
**3e**	22.20 ± 0.27	11.39 ± 2.20	94.0 ± 3.6	89.3 ± 5.4
**4a**	2.56 ± 0.52	−5.21 ± 5.22	96.7 ± 2.1	93.0 ± 3.7
**4b**	22.49 ± 1.90	0.28 ± 0.20	94.3 ± 2.9	93.3 ± 2.1
**4c**	48.73 ± 2.25	−1.32 ± 4.14	92.0 ± 4.5	93.7 ± 3.8
**4d**	14.02 ± 8.97	12.59 ± 7.43	91.3 ± 3.3	91.3 ± 2.7
**4e**	14.43 ± 0.68	5.01 ± 4.10	93.3 ± 0.5	88.0 ± 4.5
**5b**	1.35 ± 0.18	2.89 ± 4.07	95.3 ± 3.3	95.0 ± 2.9
**5c**	69.44 ± 4.39	75.01 ± 17.6	96.3 ± 2.5	90.3 ± 7.0
**5e**	62.12 ± 3.22	3.17 ± 0.78	95.7 ± 4.0	66.3 ± 3.9
**6b**	4.40 ± 1.61	34.46 ± 3.85	93.7 ± 3.1	90.3 ± 2.5
**6c**	3.19 ± 0.80	7.35 ± 1.07	91.0 ± 2.4	58.7 ± 6.1
**6e**	2.09 ± 1.86	2.19 ± 6.51	98.0 ± 1.6	97.0 ± 2.2

Results are presented as mean ± standard deviation (*n =* 3).

## Supplementary Materials

The following are available online at http://www.mdpi.com/1422-0067/19/10/3061/s1.
