# Supplementary material for: Synthesis and Biological Evaluation of Thalidomide Derivatives as Potential Anti-Psoriasis Agents
Source: Int J Mol Sci. 2018 Oct 7;19(10):3061. doi: 10.3390/ijms19103061 (PMC6212999; doi:10.3390/ijms19103061)

TKW-5651a

Pulse Sequence: s2pul
UNITYplus-400 "unity400"
Date: Oct 14 2016
Solvent: DMSO
Ambient temperature
Total 64 repetitions

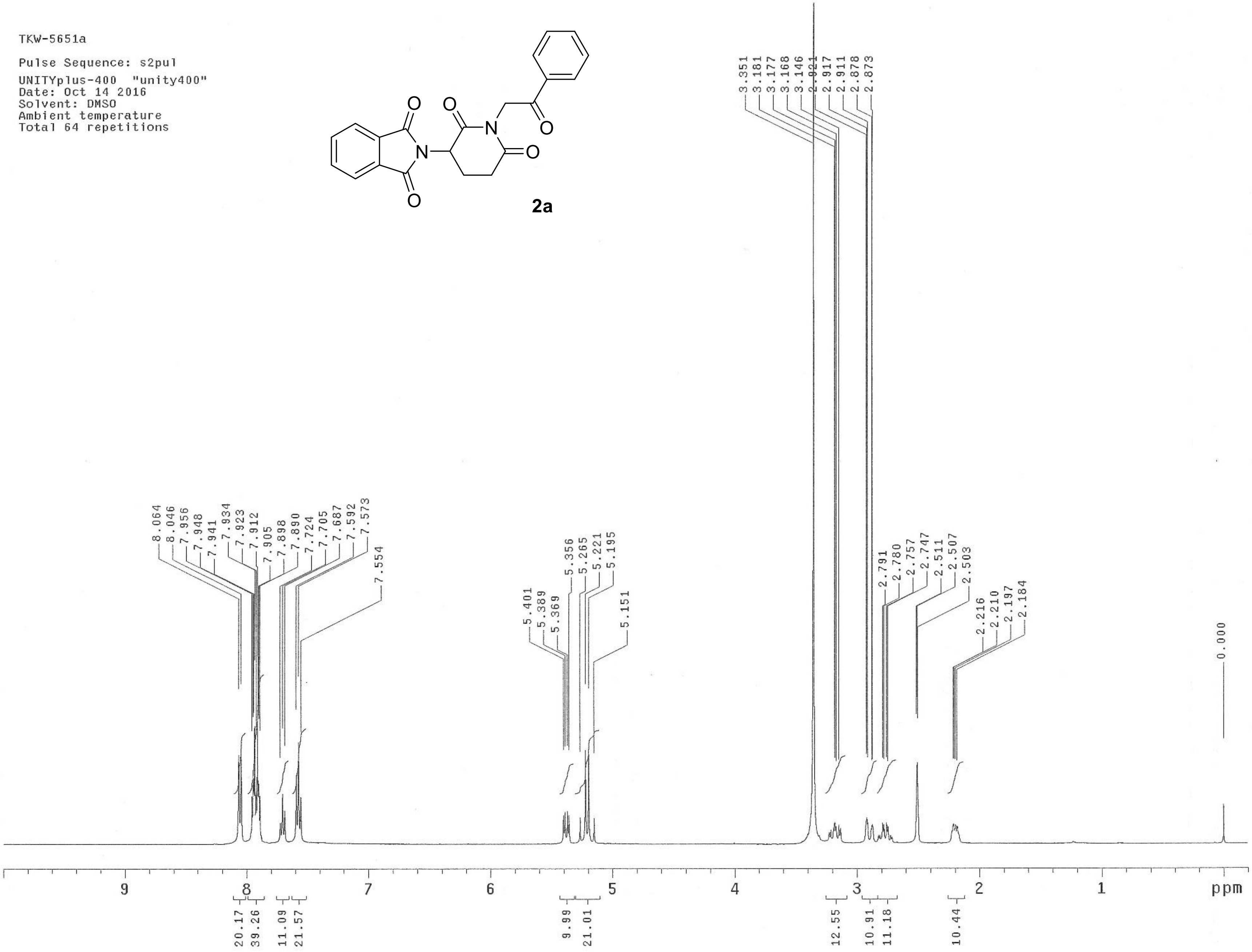

TKW-5651a

Pulse Sequence: s2pul

UNITYplus-400 "unity400"
Date: Oct 14 2016
Solvent: DMSO
Ambient temperature
Total 2080 repetitions

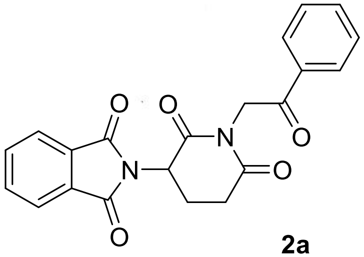

**2a**

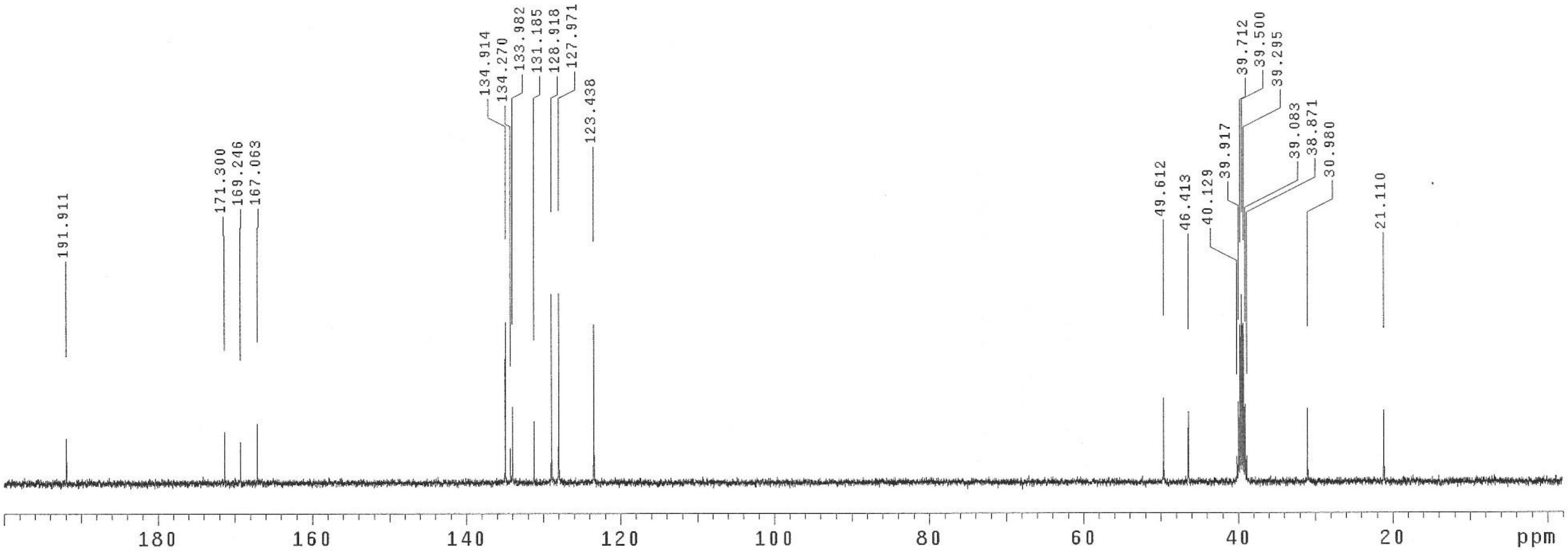

TKW-5652b

Pulse Sequence: s2pul
UNITYplus-400 "unity400"
Date: Oct 14 2016
Solvent: DMSO
Ambient temperature
Total 32 repetitions

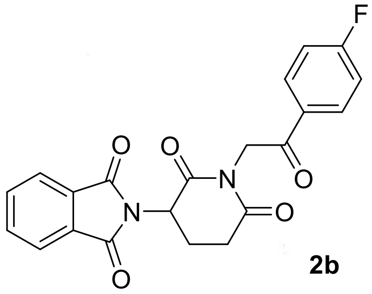


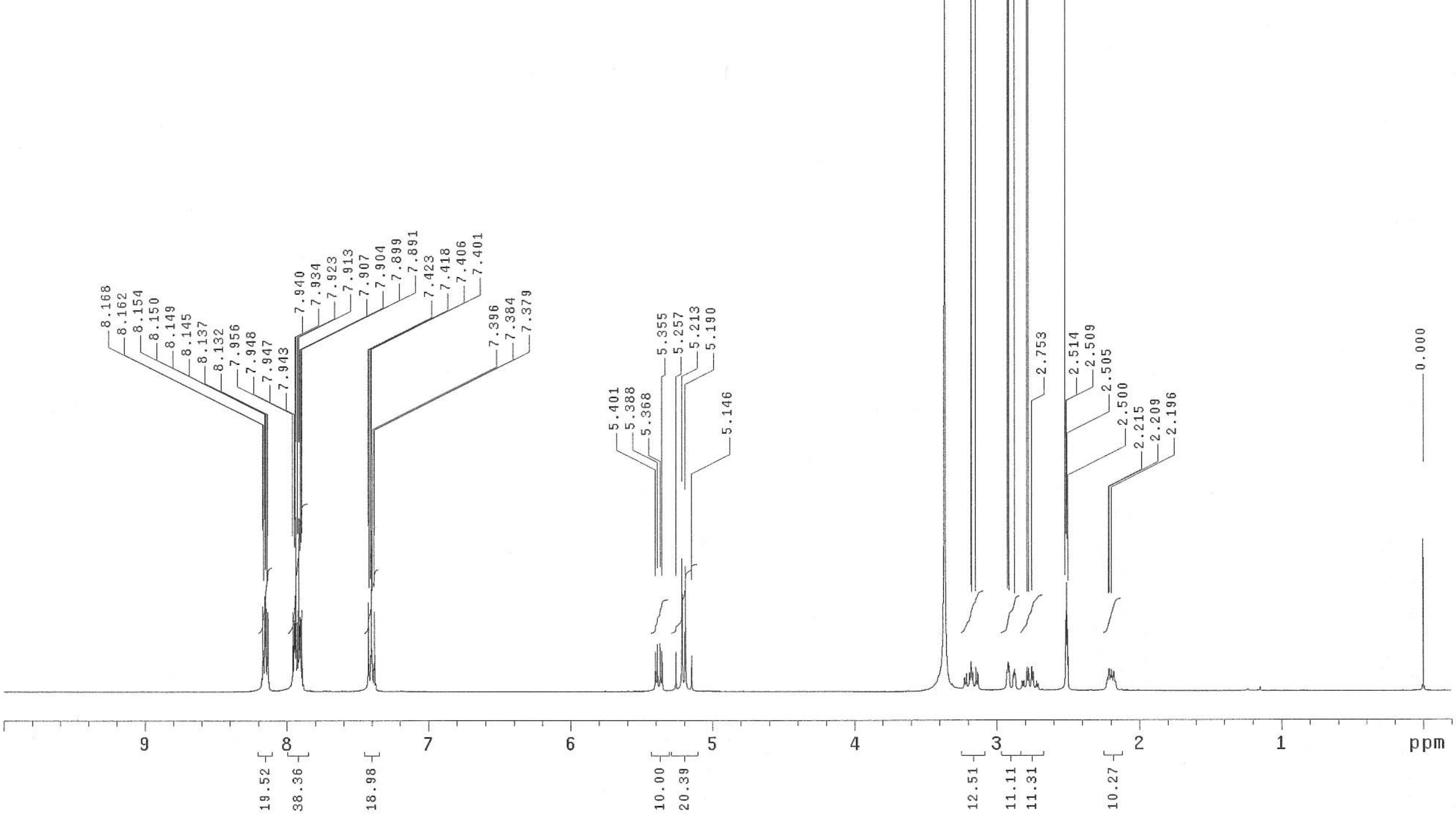

TKW-5652b

Pulse Sequence: s2pul
UNITYplus-400 "unity400"
Date: Oct 14 2016
Solvent: DMSO
Ambient temperature
Total 3264 repetitions

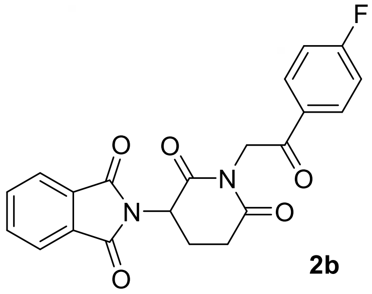

**2b**

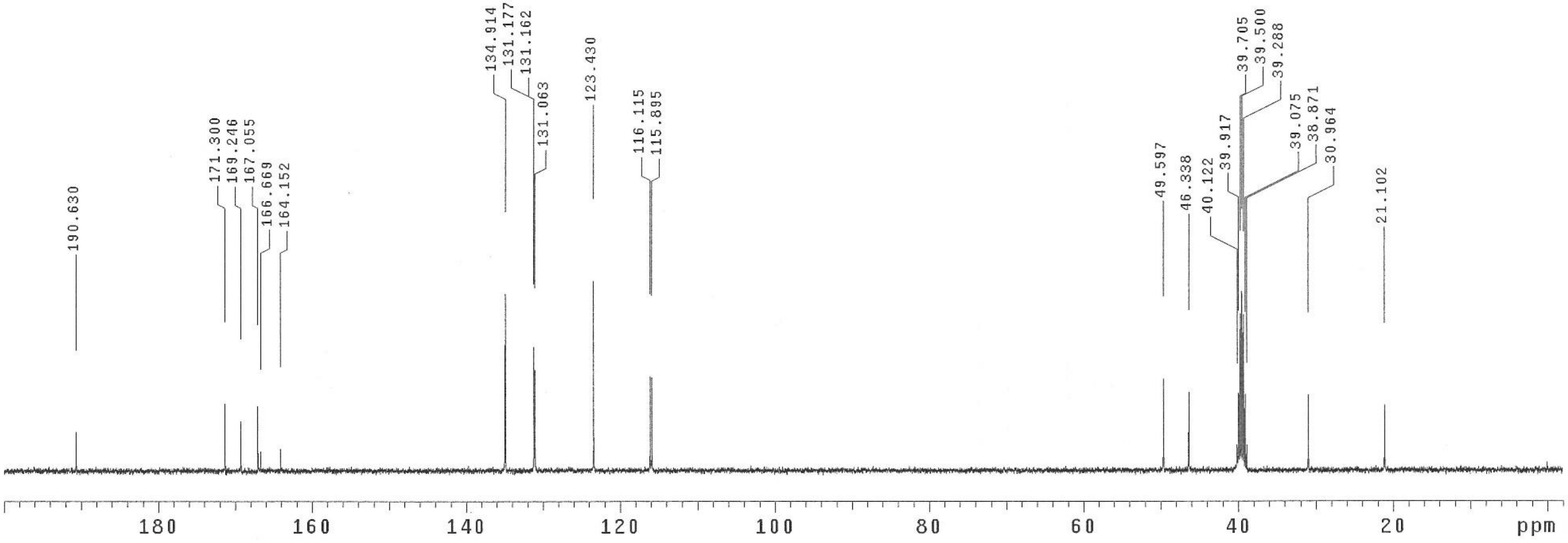

TKW-5683g

Pulse Sequence: s2pul
UNITYplus-400 "unity400"
Date: Nov 15 2016
Solvent: DMSO
Ambient temperature
Total 64 repetitions

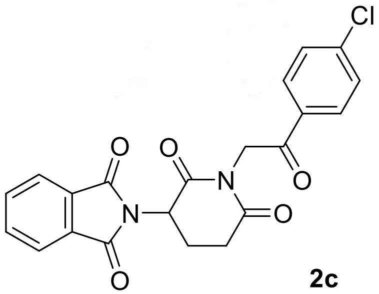


**2c**

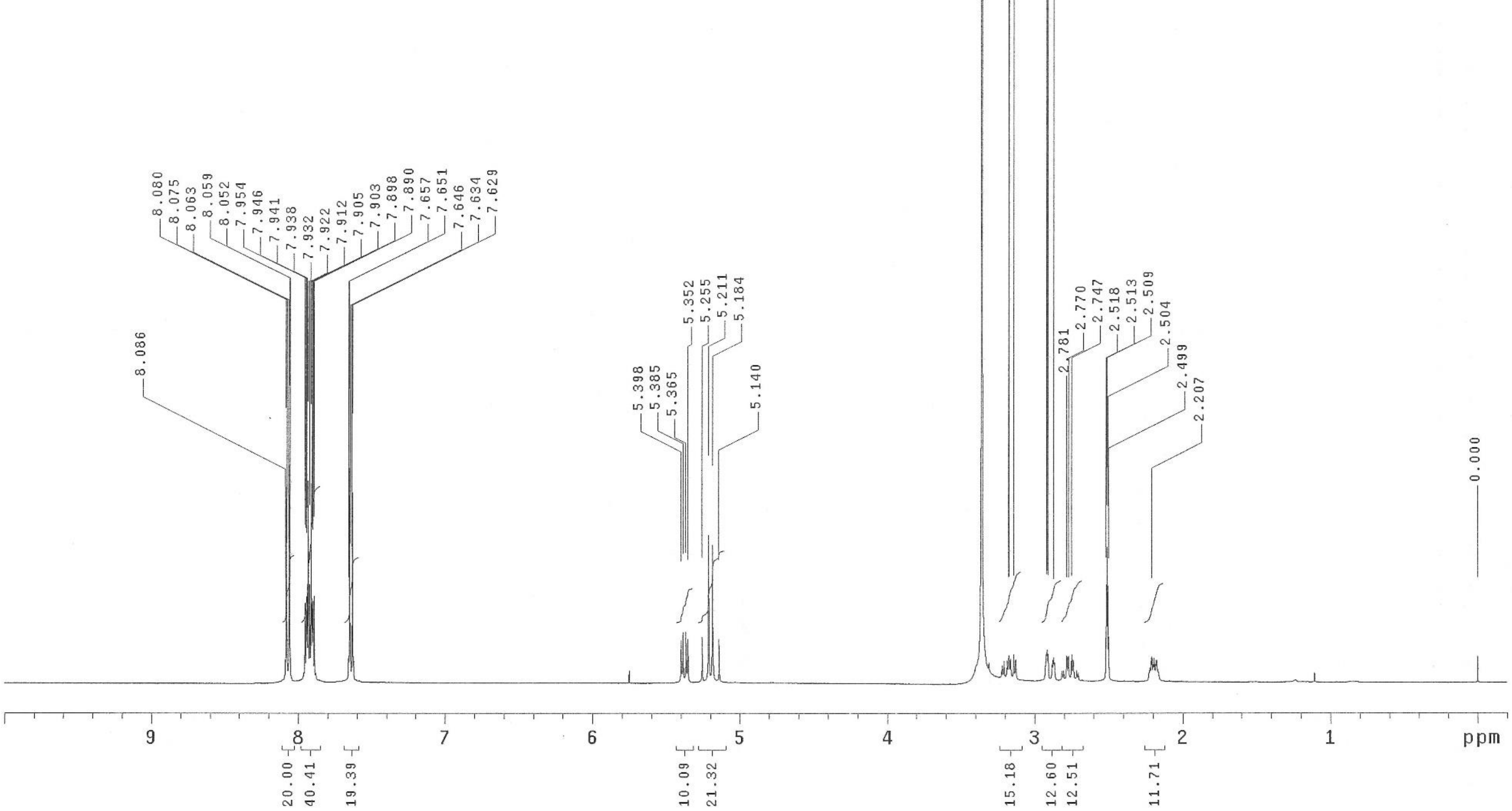

TKW-5683g

Pulse Sequence: s2pul

UNITYplus-400 "unity400"
Date: Nov 15 2016
Solvent: DMSO
Ambient temperature
Total 4736 repetitions

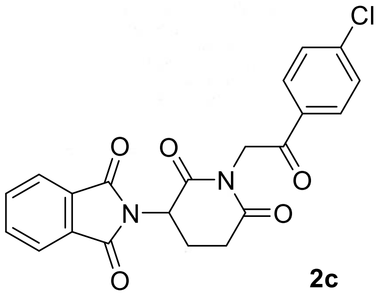


**2c**

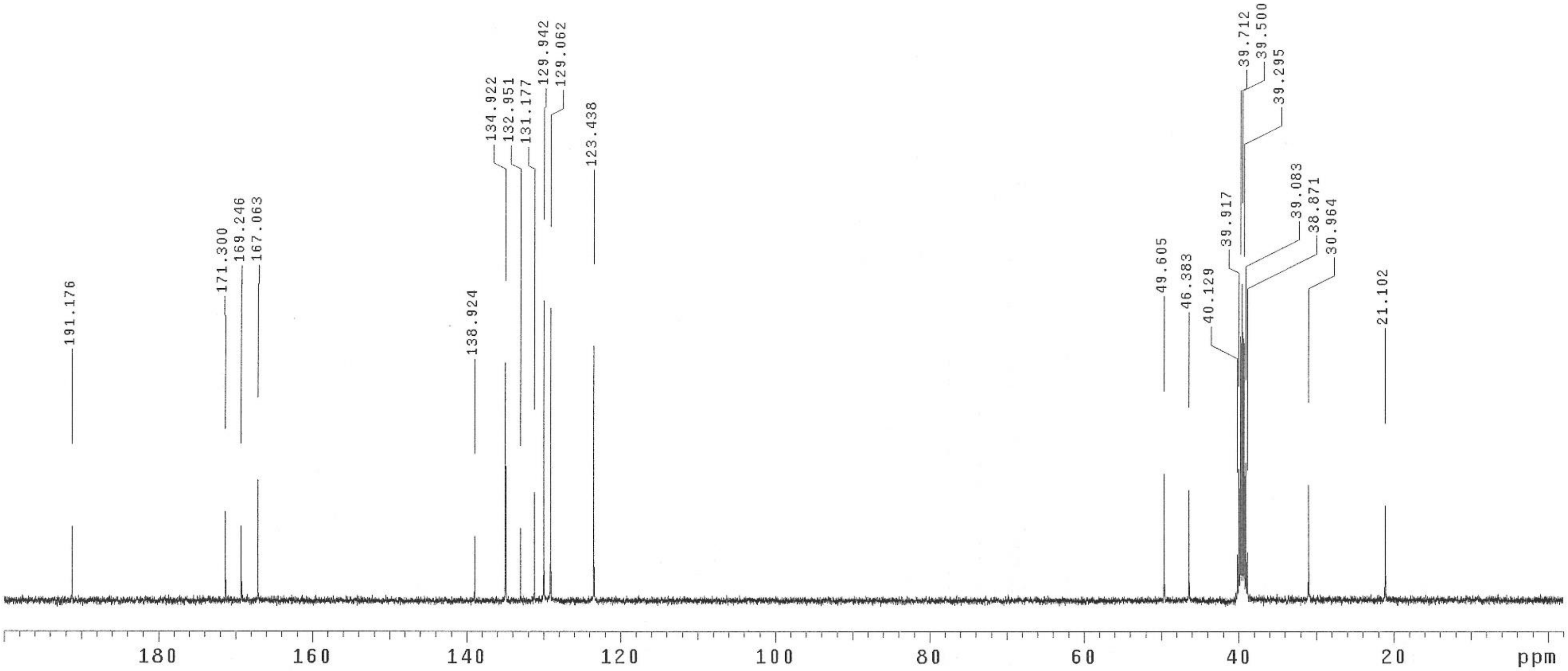

TKW-5661k

Pulse Sequence: s2pul
UNITYplus-400 "unity400"
Date: Jun 2 2016
Solvent: DMSO
Ambient temperature
Total 64 repetitions

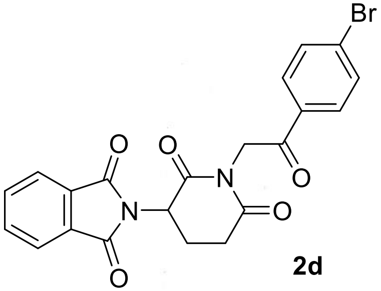

**2d**

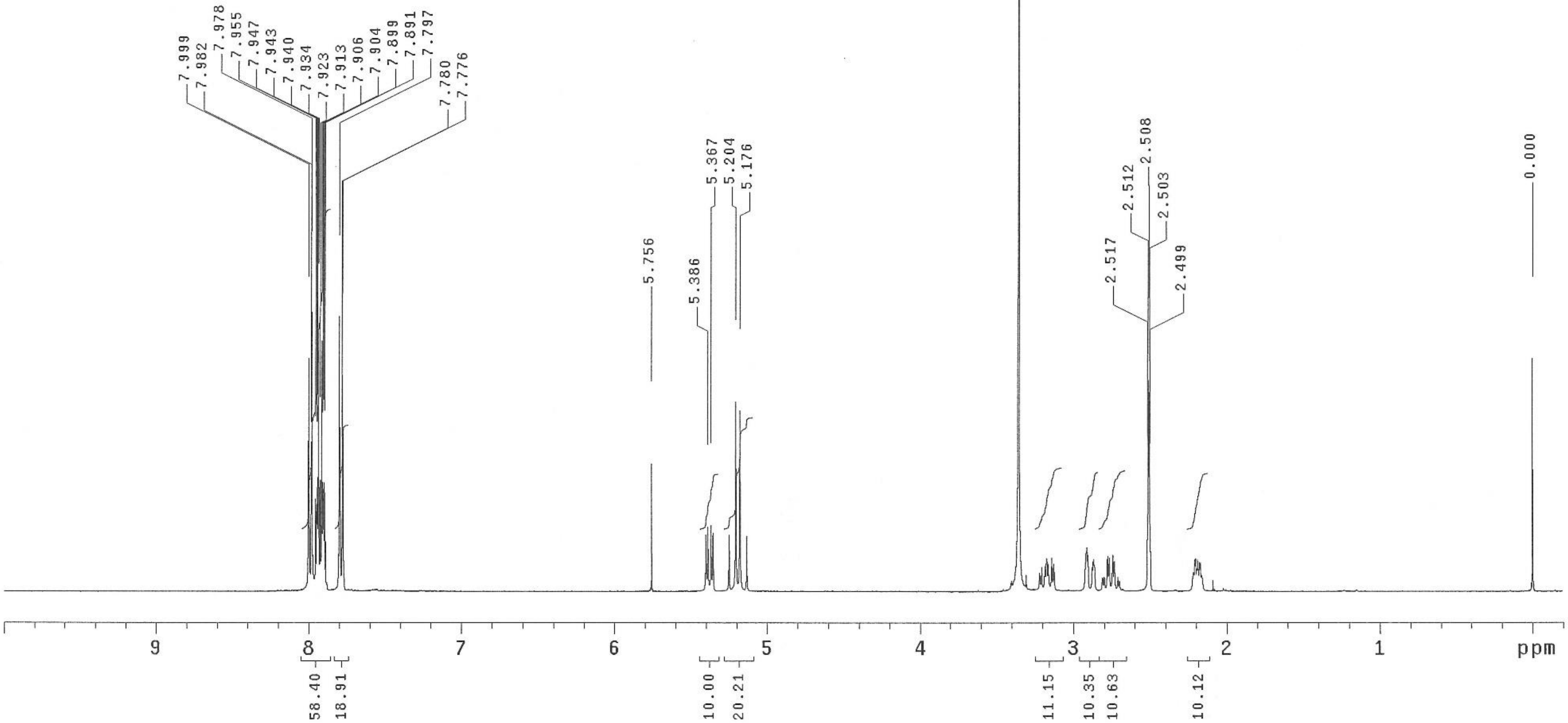

TKW-5661k

Pulse Sequence: s2pul
UNITYplus-400 "unity400"
Date: Jun 2 2016
Solvent: DMSO
Ambient temperature
Total 1664 repetitions

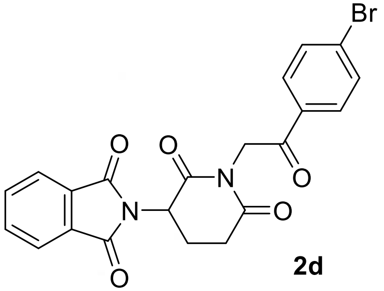


**2d**

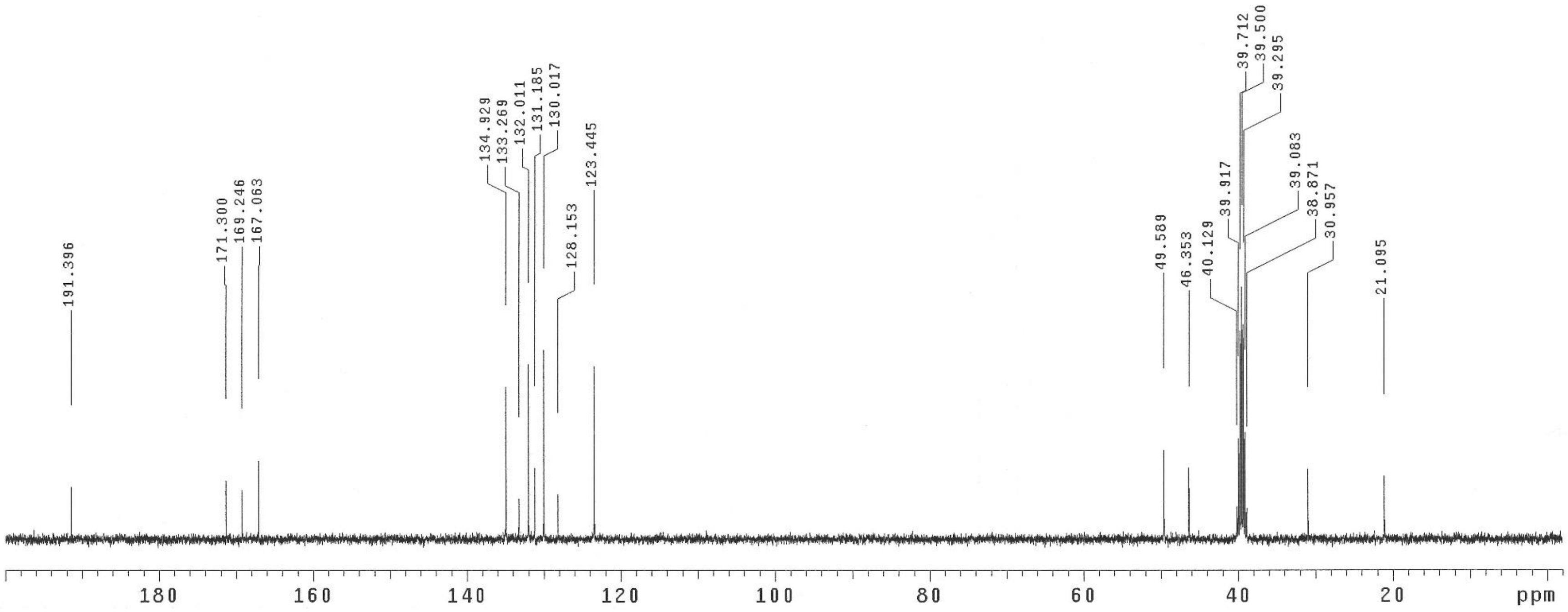

TKW-5653c

Pulse Sequence: s2pul
UNITYplus-400 "unity400"
Date: Oct 14 2016
Solvent: DMSO
Ambient temperature
Total 32 repetitions

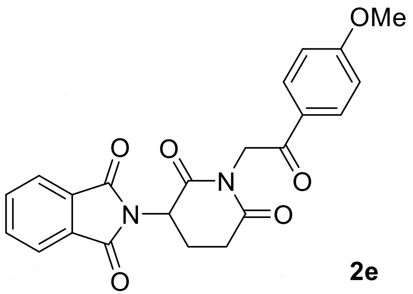


**2e**

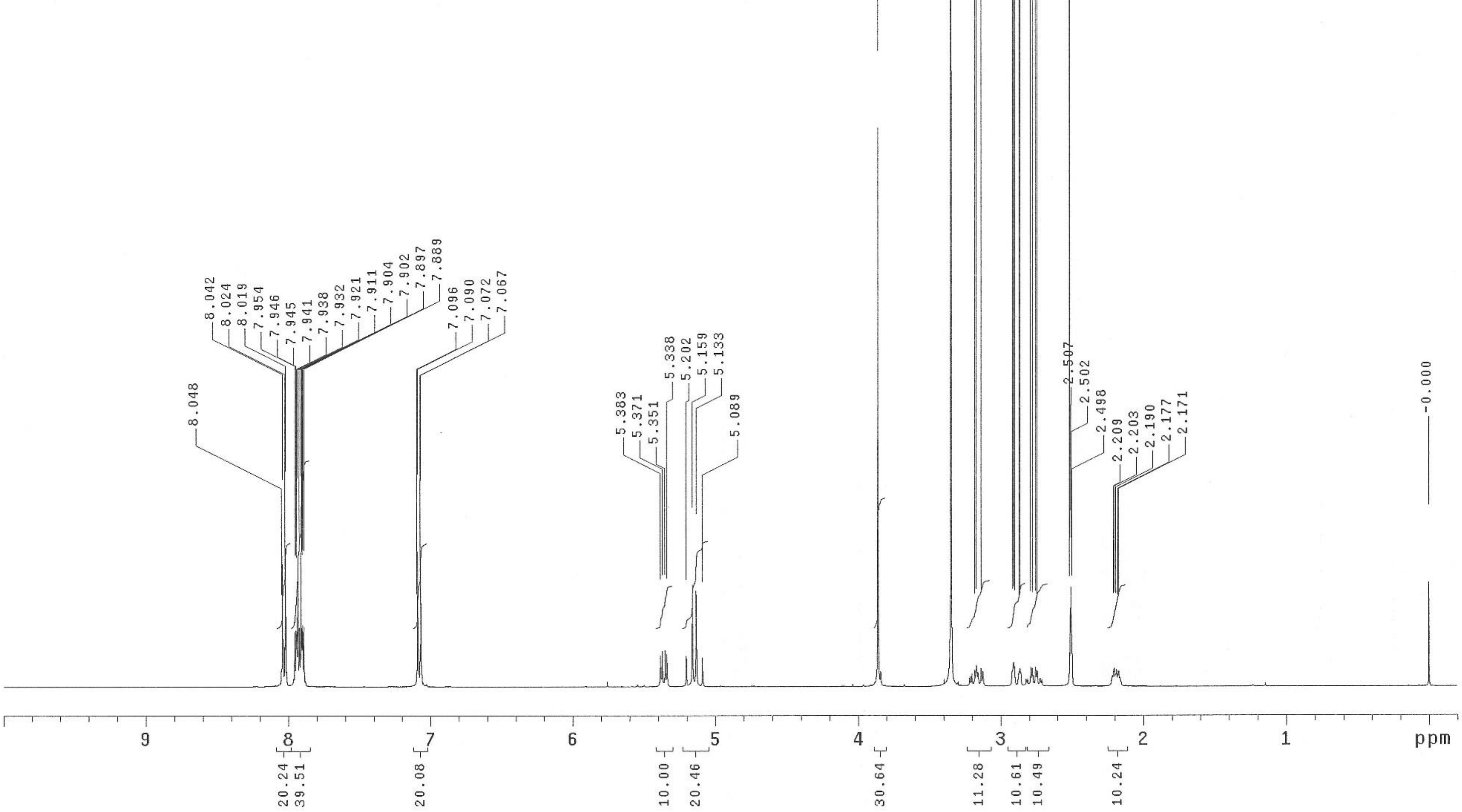

TKW-5653c

Pulse Sequence: s2pul
UNITYplus-400 "unity400"
Date: Oct 14 2016
Solvent: DMSO
Ambient temperature
Total 2768 repetitions

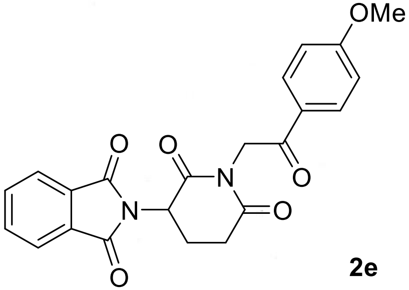


2e

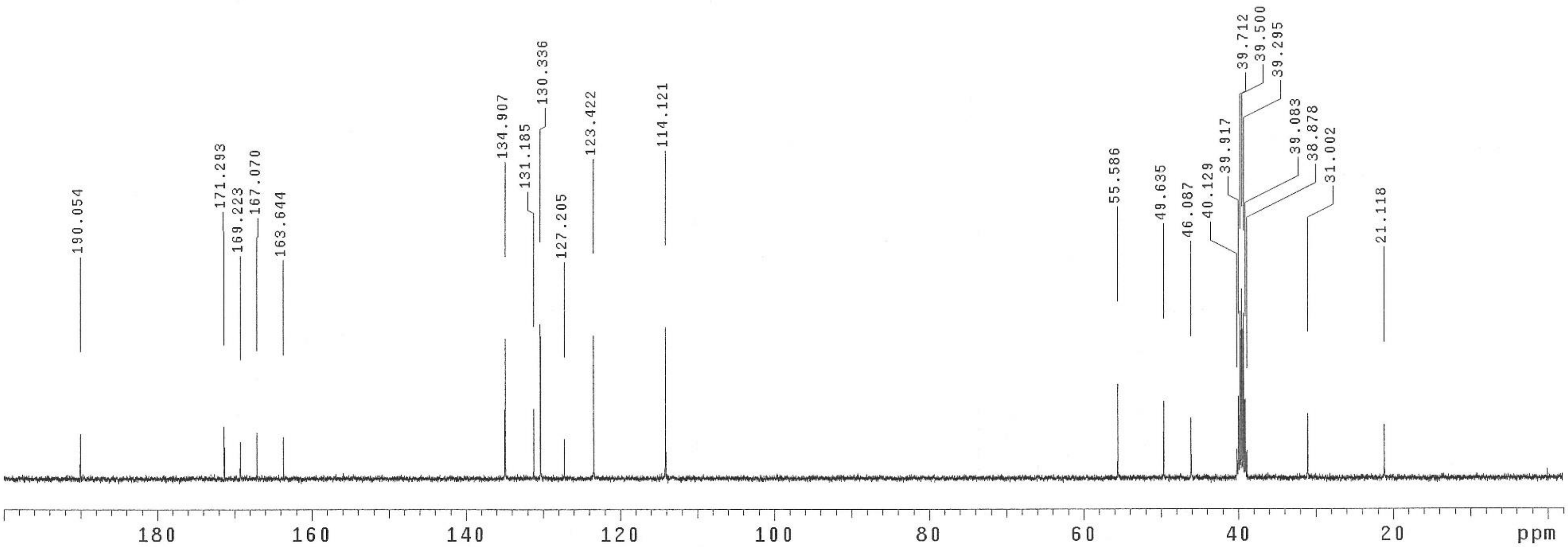

TKW-5917q

Pulse Sequence: s2pul
UNITYplus-400 "unity400"
Date: Dec 18 2017
Solvent: DMSO
Ambient temperature
Total 64 repetitions

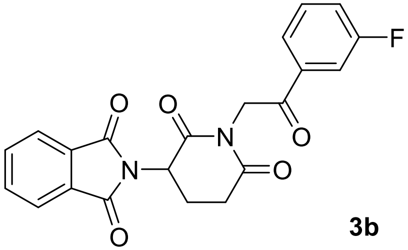


**3b**

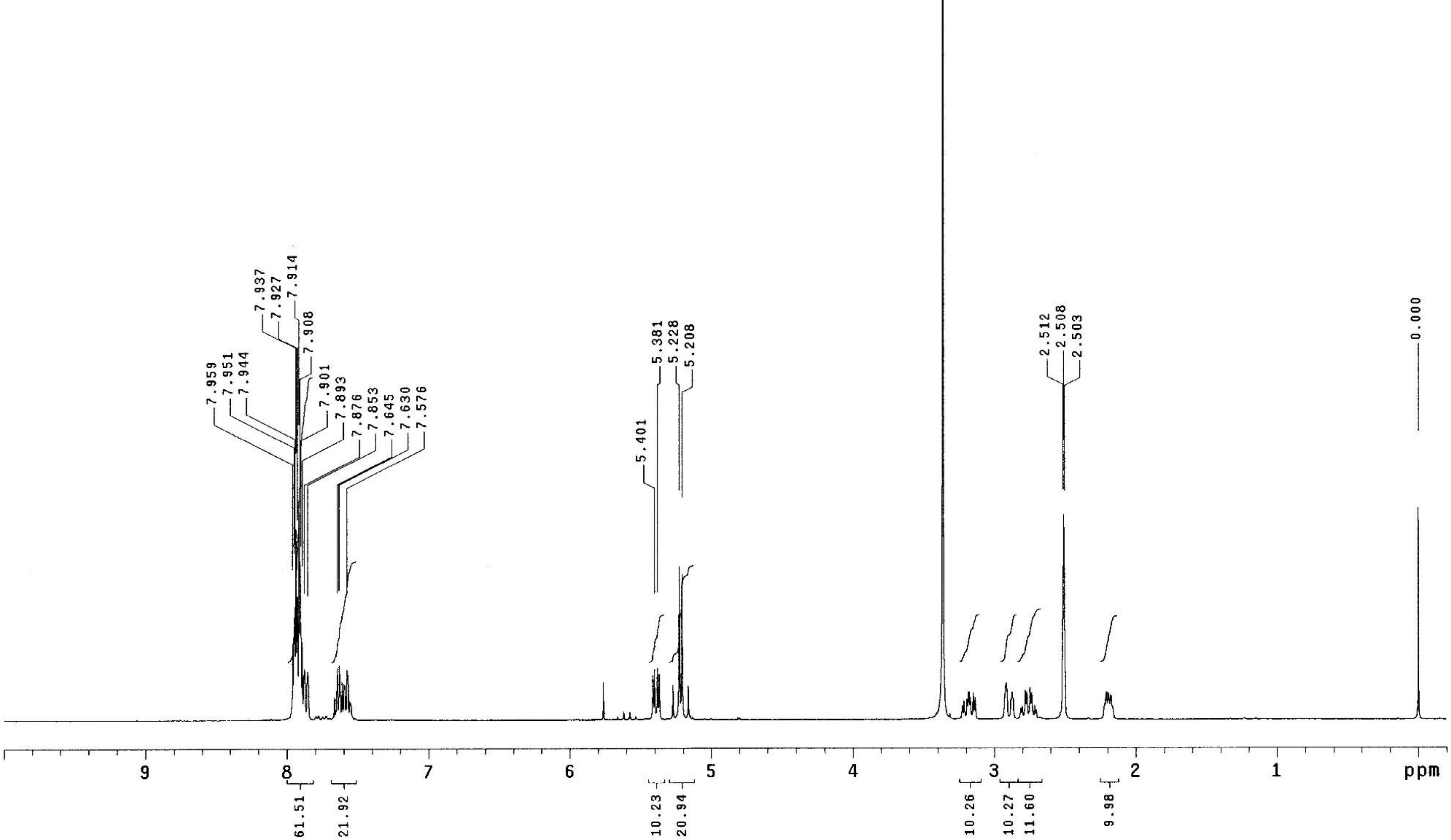

TKW-5917q

Pulse Sequence: s2pul
UNITYplus-400 "unity400"
Date: Dec 18 2017
Solvent: DMSO
Ambient temperature
Total 5440 repetitions

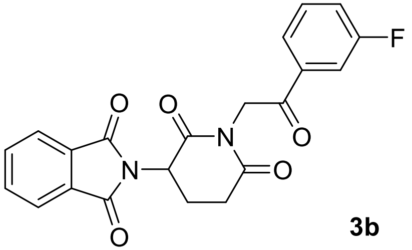


3b

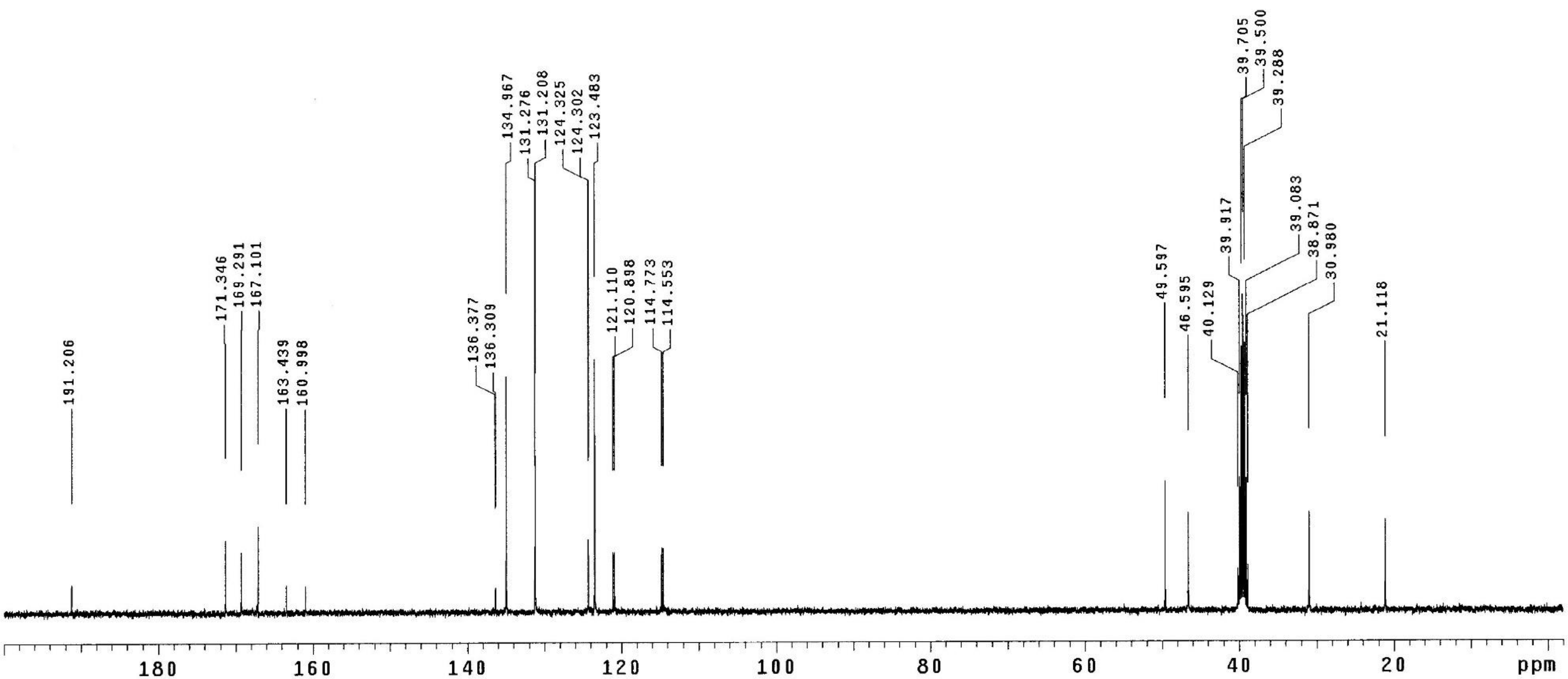

TKW-5694r

Pulse Sequence: s2pul
UNITYplus-400 "unity400"
Date: Apr 27 2017
Solvent: DMSO
Ambient temperature
Total 80 repetitions

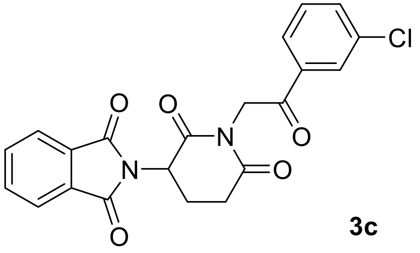

**3c**

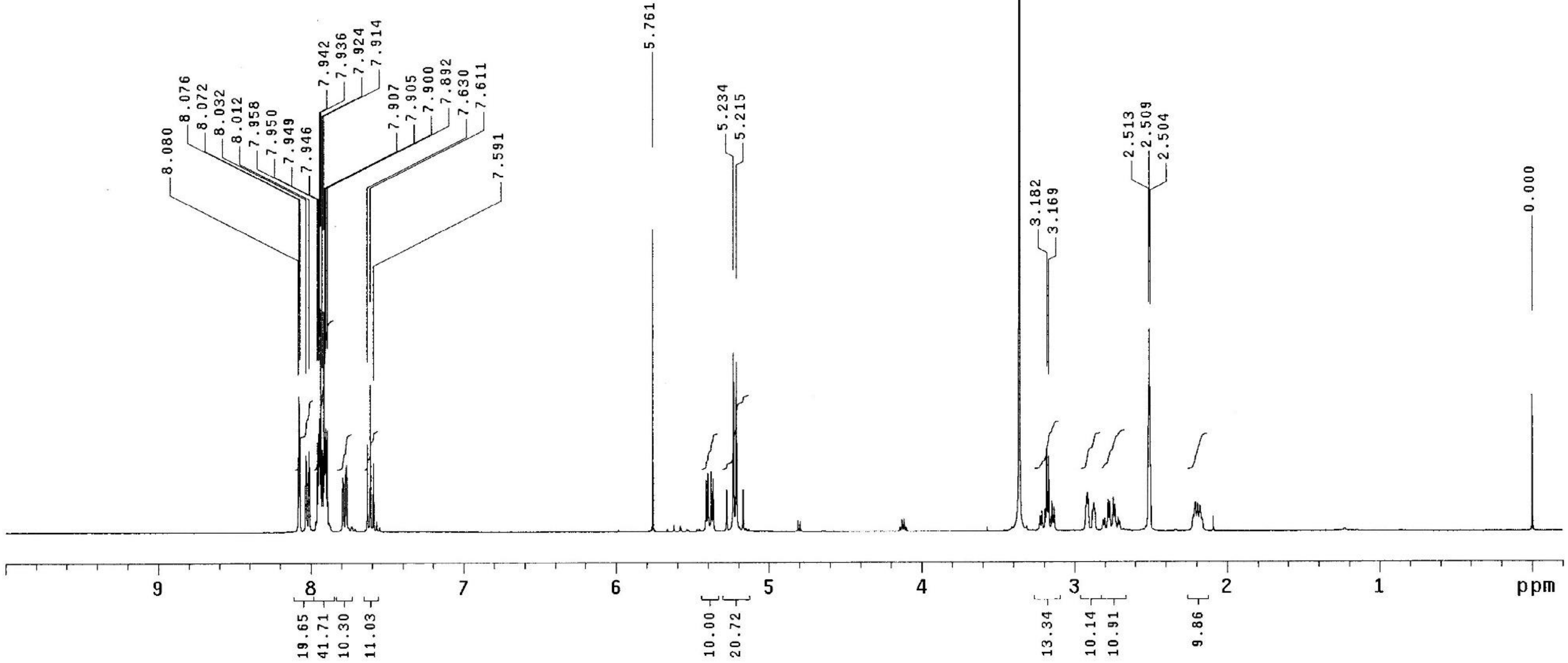

TKW-5694r

Pulse Sequence: s2pul
UNITYplus-400 "unity400"
Date: Apr 27 2017
Solvent: DMSO
Ambient temperature
Total 16000 repetitions

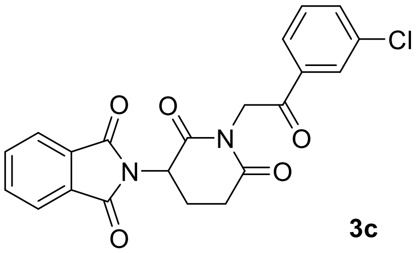


**3c**

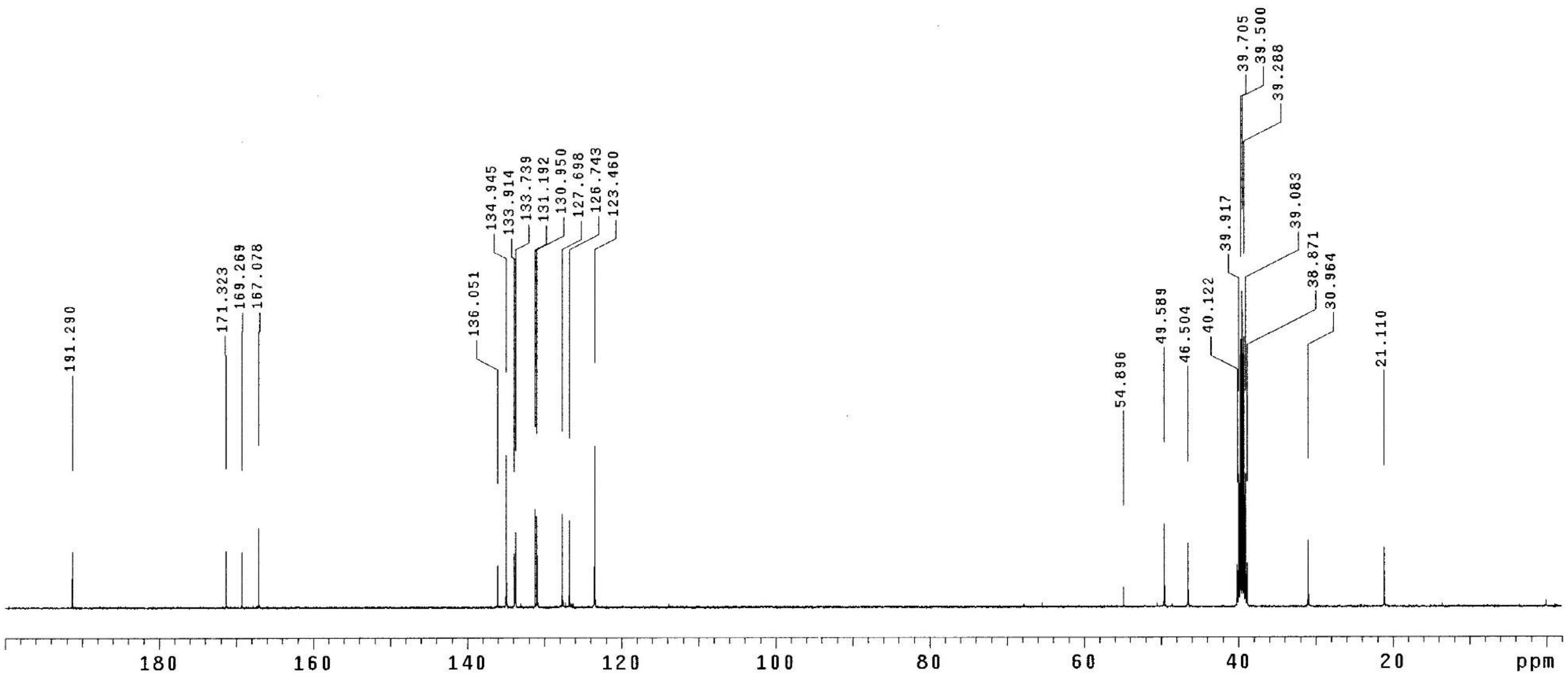

TKW-5660j

Pulse Sequence: s2pul
UNITYplus-400 "unity400"
Date: Jun 2 2016
Solvent: DMSO
Ambient temperature
Total 32 repetitions

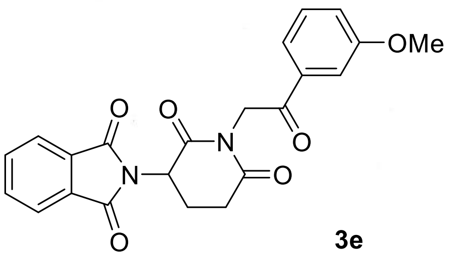


3e

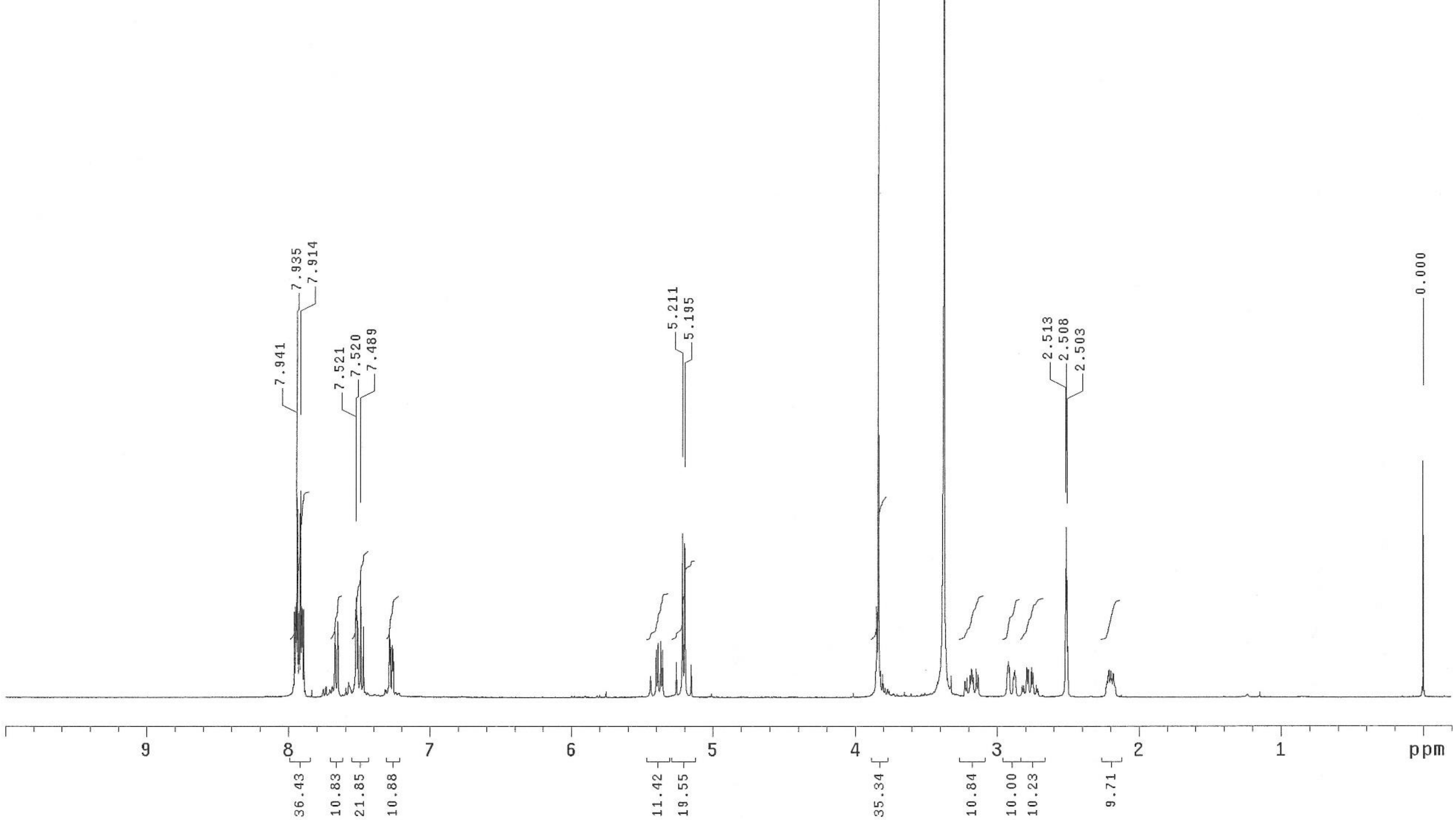

TKW-5660j

Pulse Sequence: s2pul
UNITYplus-400 "unity400"
Date: Jun 2 2016
Solvent: DMSO
Ambient temperature
Total 2224 repetitions

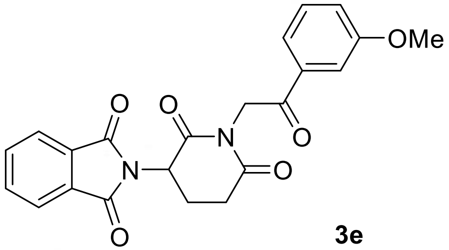


3e

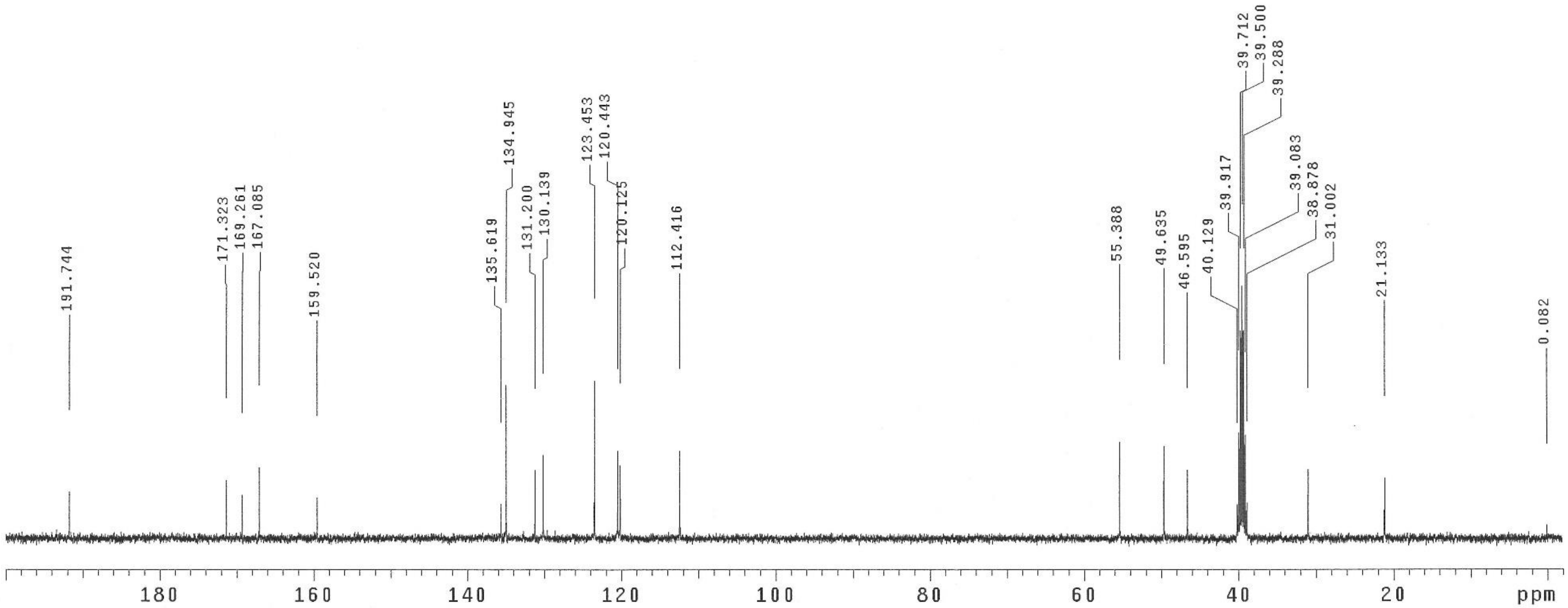

TKW-5656f

Pulse Sequence: s2pul
UNITYplus-400 "unity400"
Date: Oct 17 2016
Solvent: DMSO
Ambient temperature
Total 32 repetitions

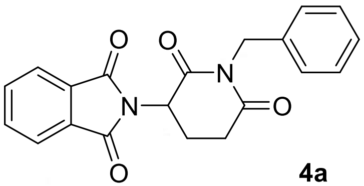

4a

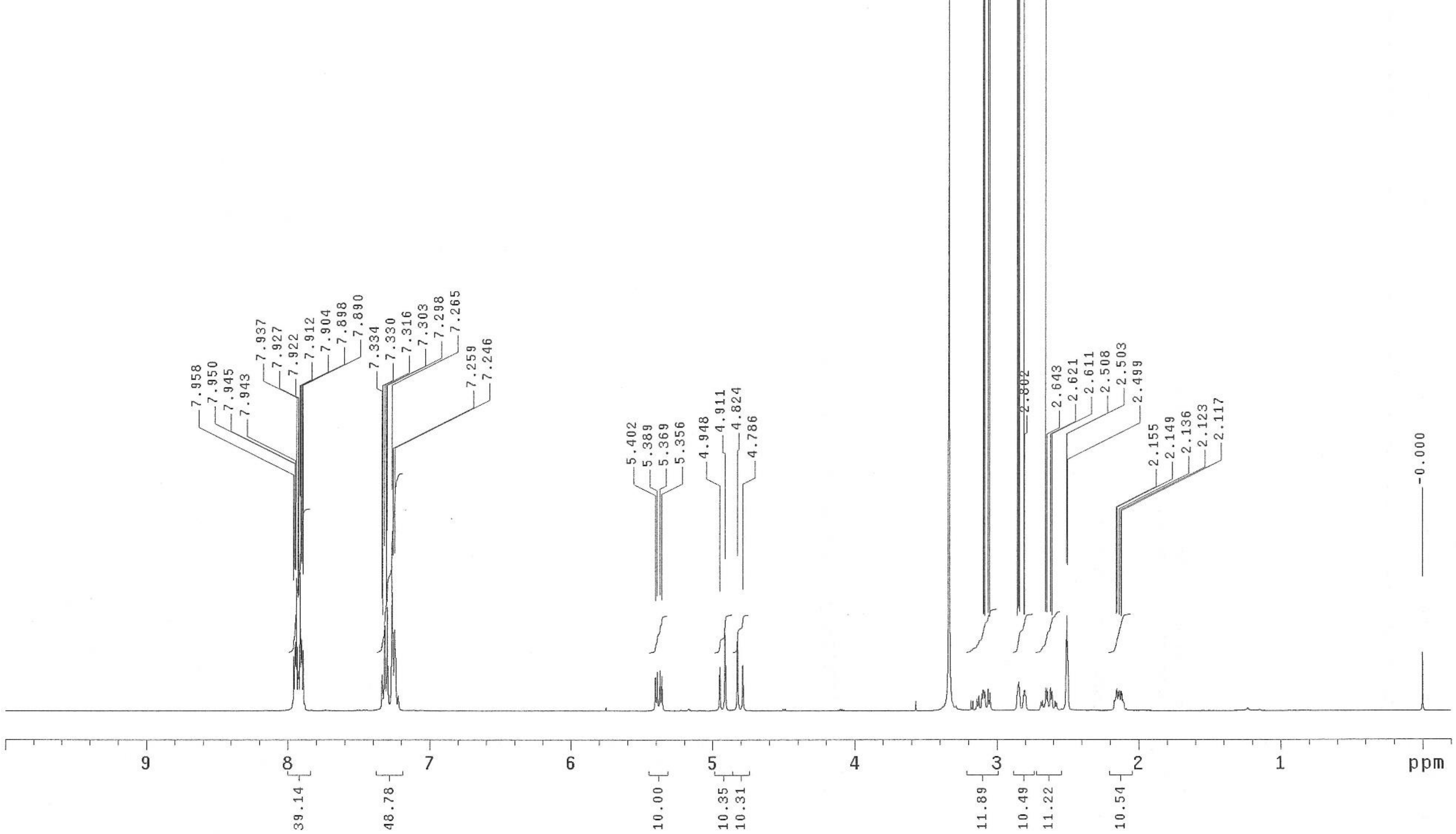

TKW-5656f

Pulse Sequence: s2pul

UNITYplus-400 "unity400"
Date: Oct 17 2016
Solvent: DMSO
Ambient temperature
Total 2576 repetitions

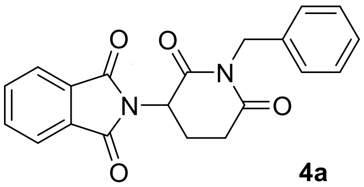

4a

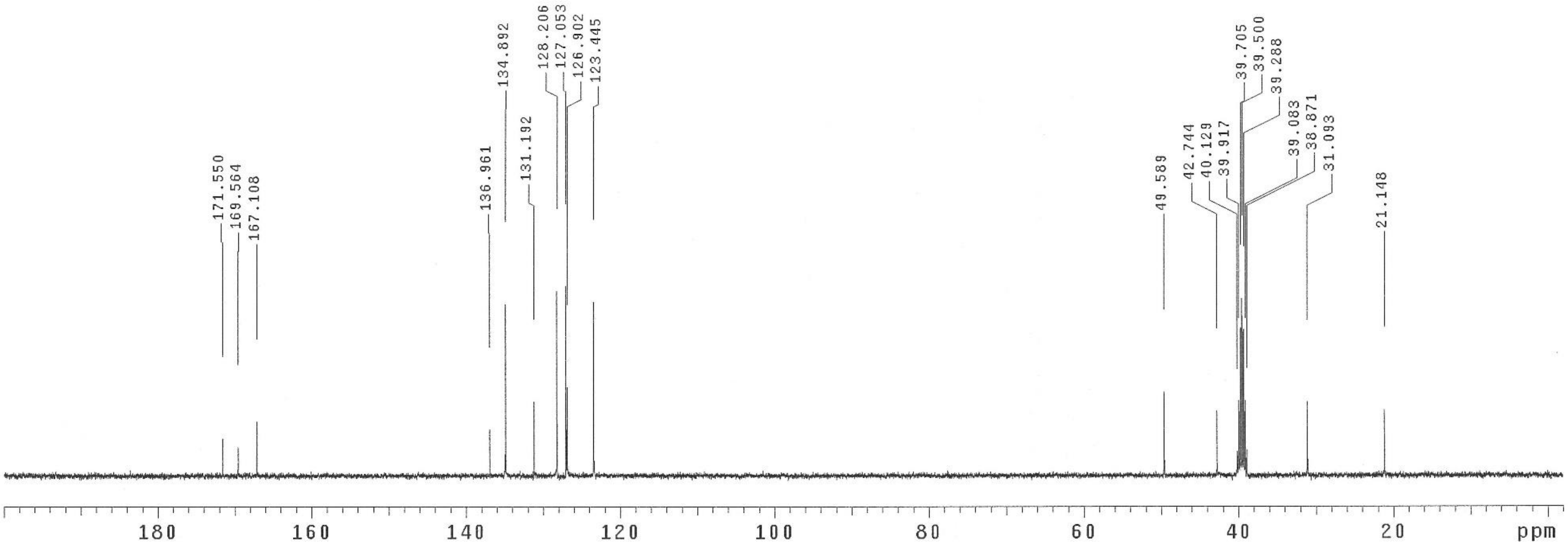

TKW-5658h

Pulse Sequence: s2pul
UNITYplus-400 "unity400"
Date: Jun 2 2016
Solvent: DMSO
Ambient temperature
Total 64 repetitions

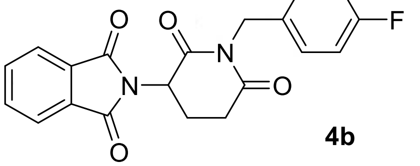


**4b**

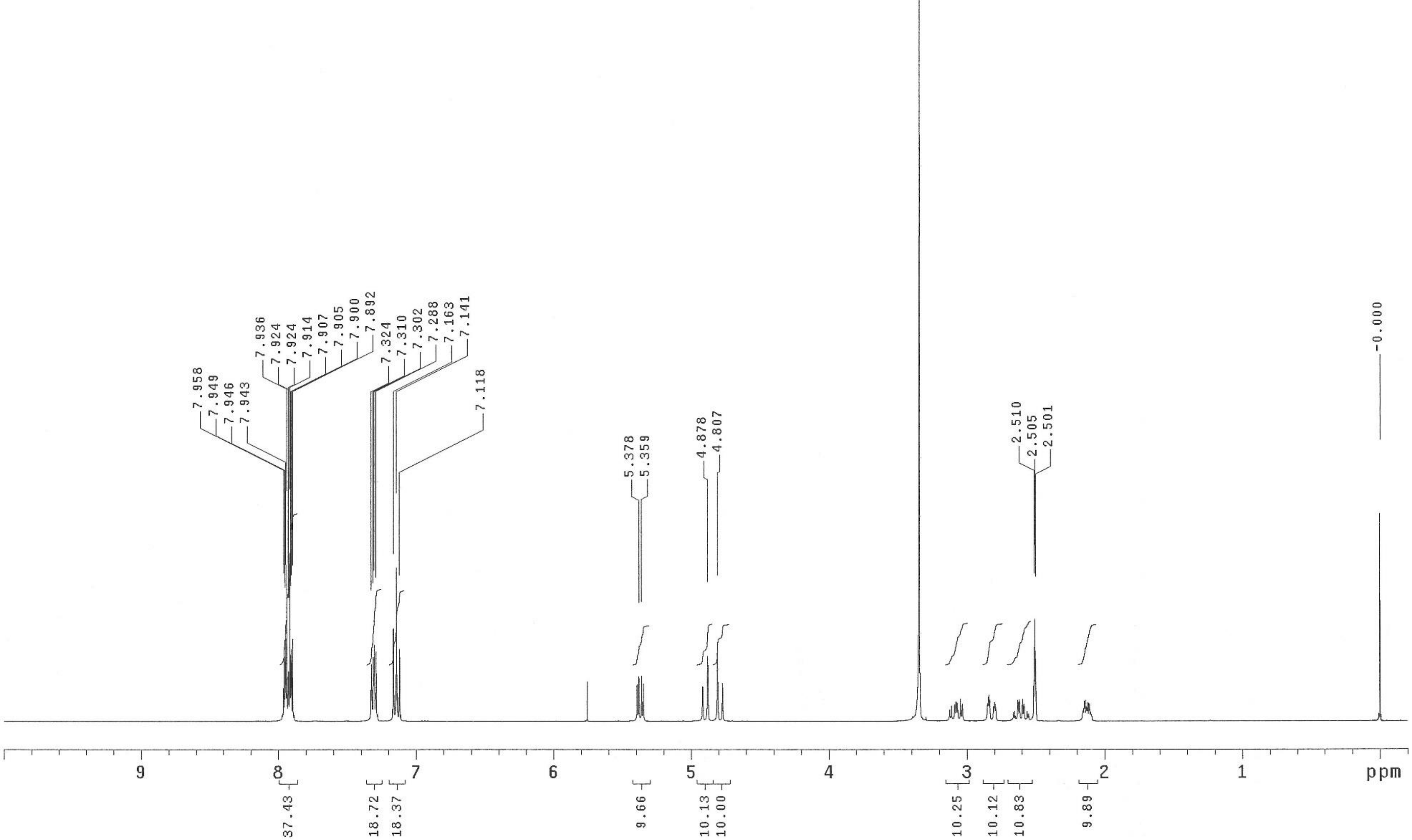

TKW-5658h

Pulse Sequence: s2pul
UNITYplus-400 "unity400"
Date: Jun 2 2016
Solvent: DMSO
Ambient temperature
Total 4144 repetitions

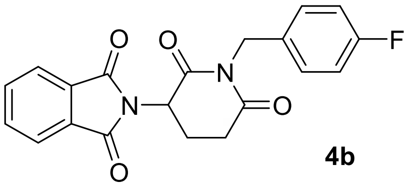

**4b**

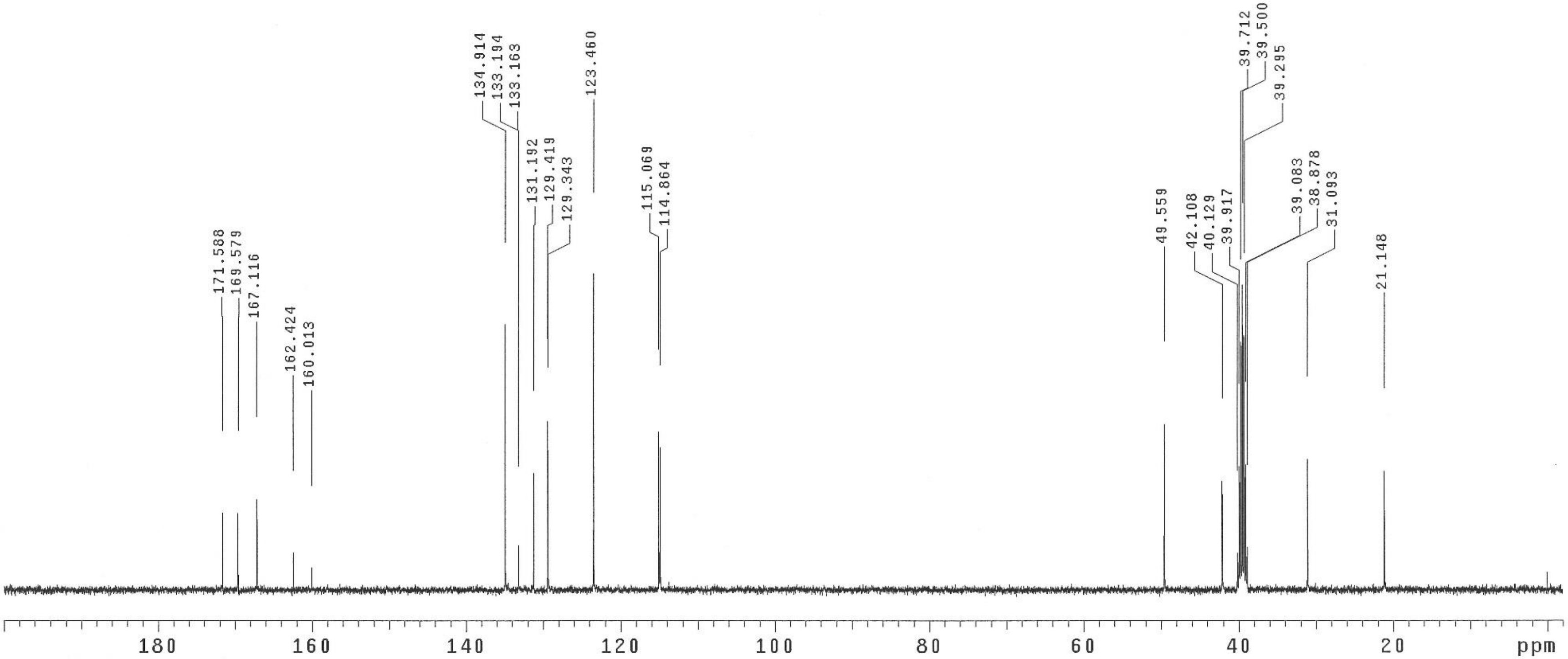

TKW-5681e

Pulse Sequence: s2pul
UNITYplus-400 "unity400"
Date: Oct 24 2016
Solvent: DMSO
Ambient temperature
Total 32 repetitions

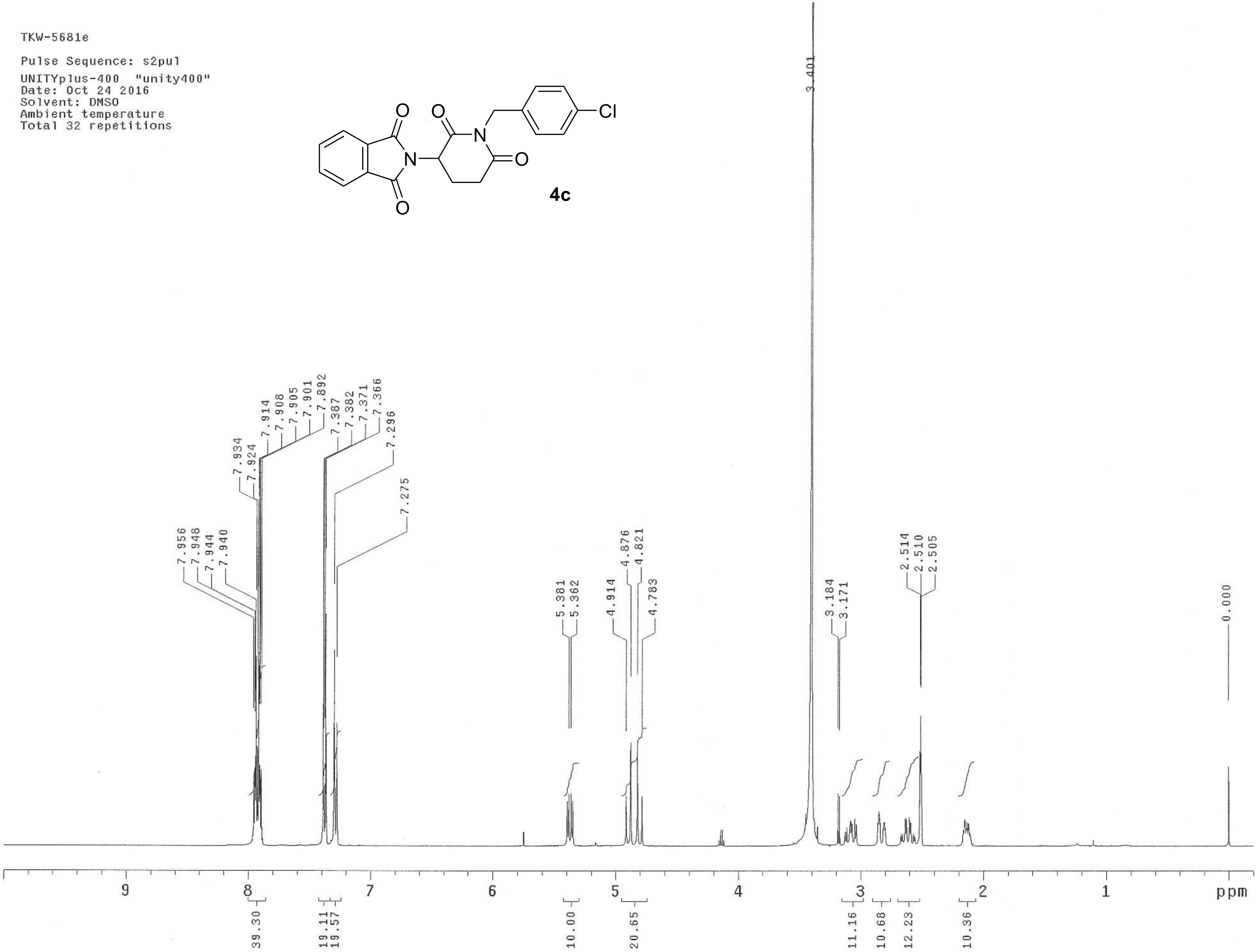

TKW-5681e

Pulse Sequence: s2pul
UNITYplus-400 "unity400"
Date: Oct 24 2016
Solvent: DMSO
Ambient temperature
Total 2512 repetitions

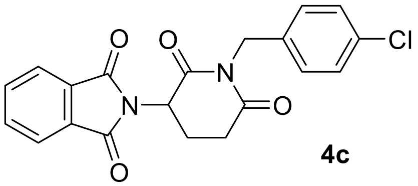


**4c**

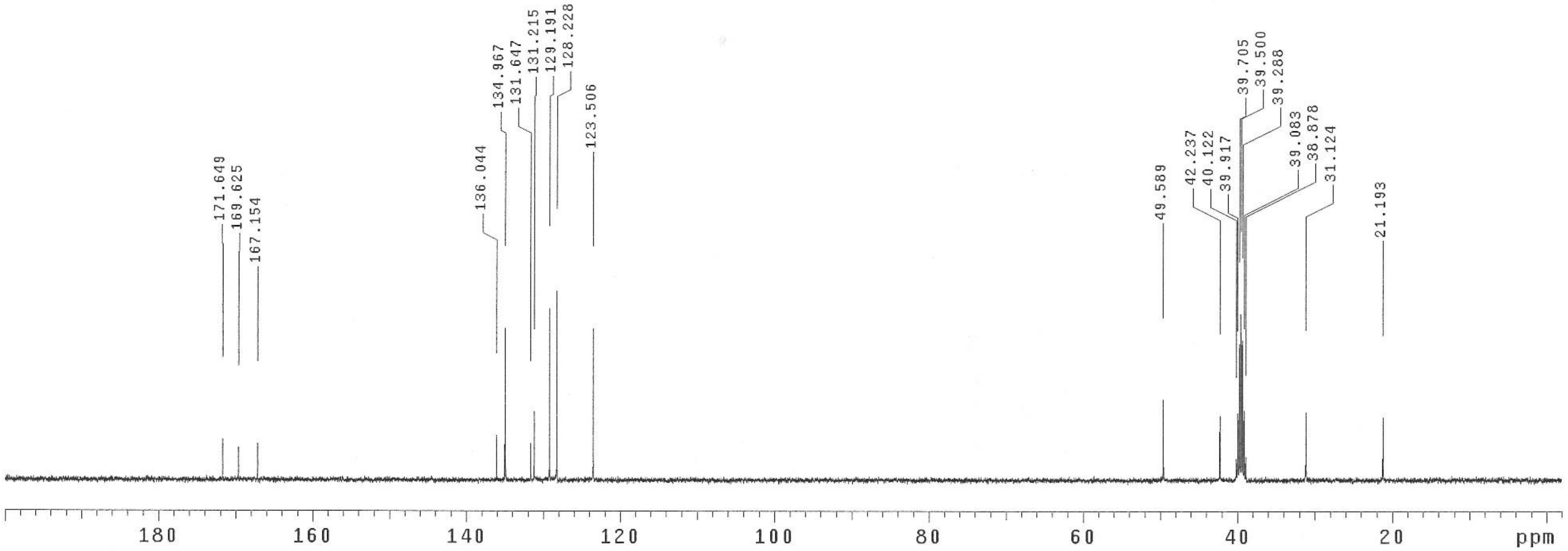

TKW-5915o

Pulse Sequence: s2pul
Mercury-400BB "MerPlus400"
Date: Dec 6 2017
Solvent: dmso
Ambient temperature
Total 64 repetitions

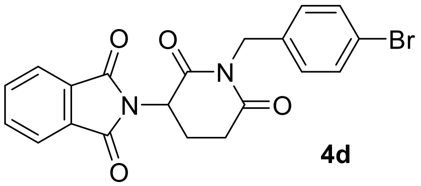


**4d**

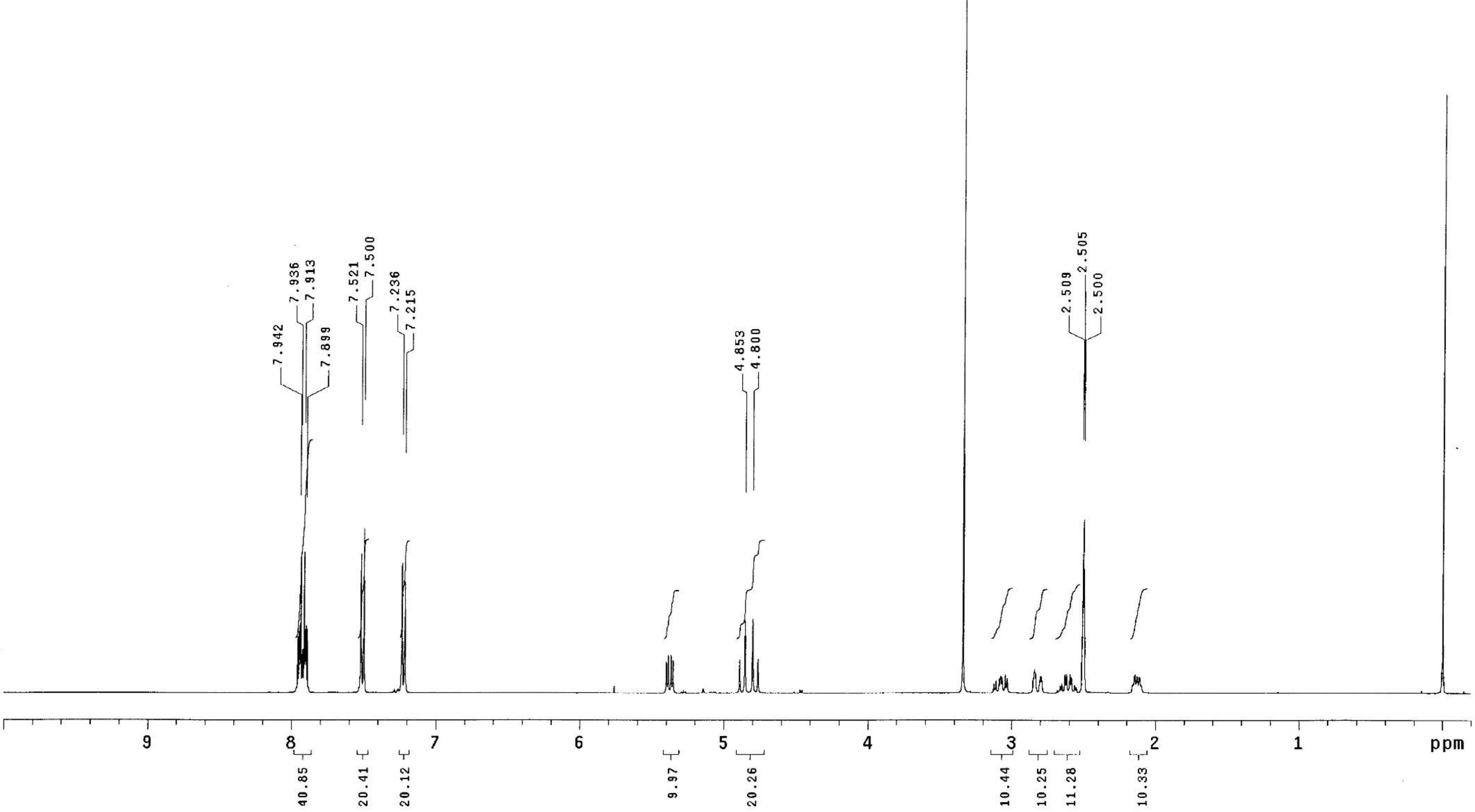

TKW-5915o

Pulse Sequence: s2pul
Mercury-400BB "MerPlus400"
Date: Dec 6 2017
Solvent: dmso
Ambient temperature
Total 3824 repetitions

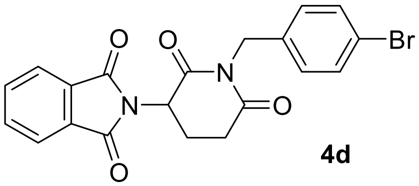


**4d**

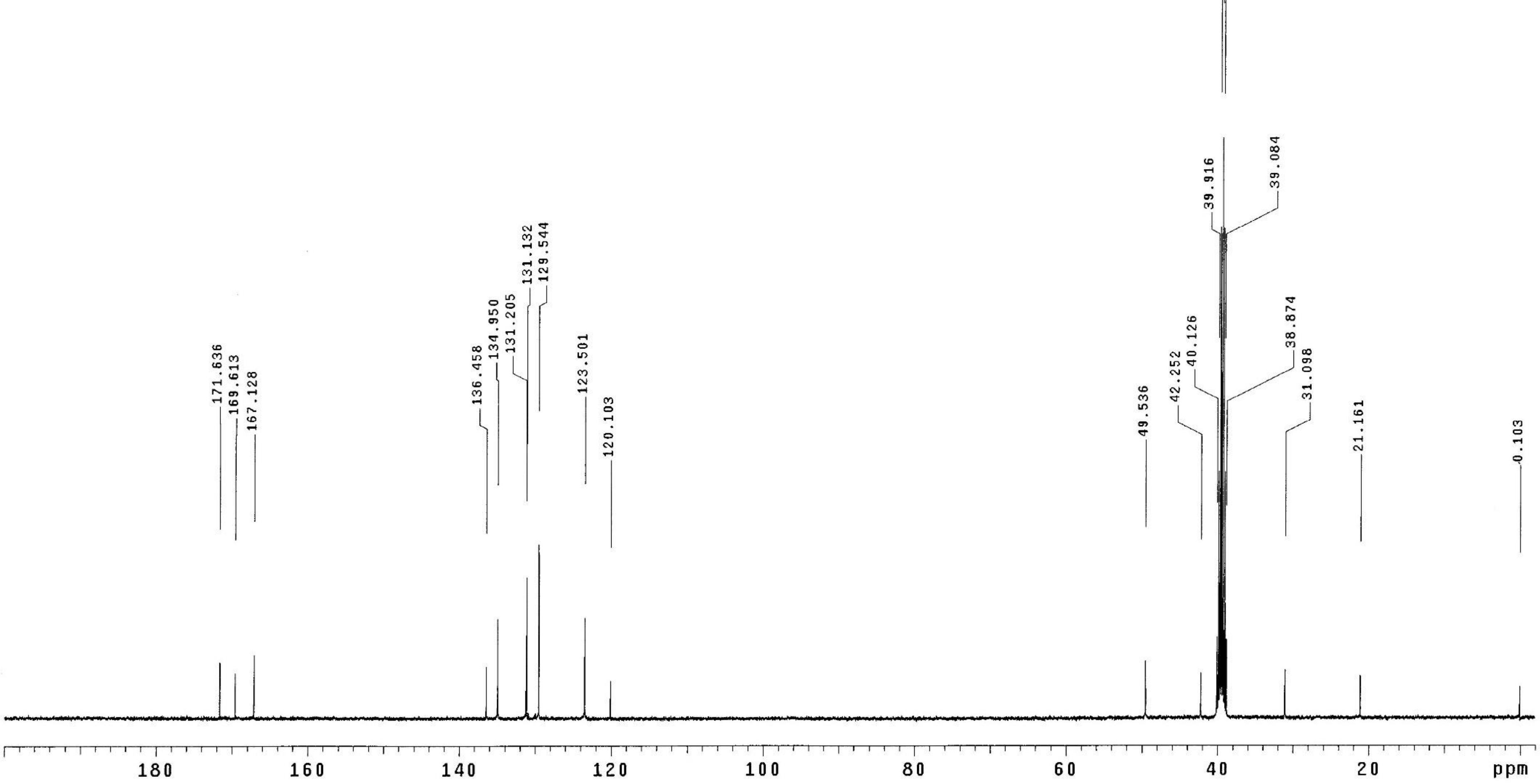

TKW-5659i

Pulse Sequence: s2pul
UNITYplus-400 "unity400"
Date: Aug 17 2016
Solvent: DMSO
Ambient temperature
Total 64 repetitions

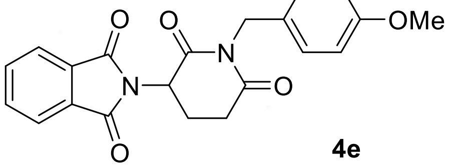


**4e**

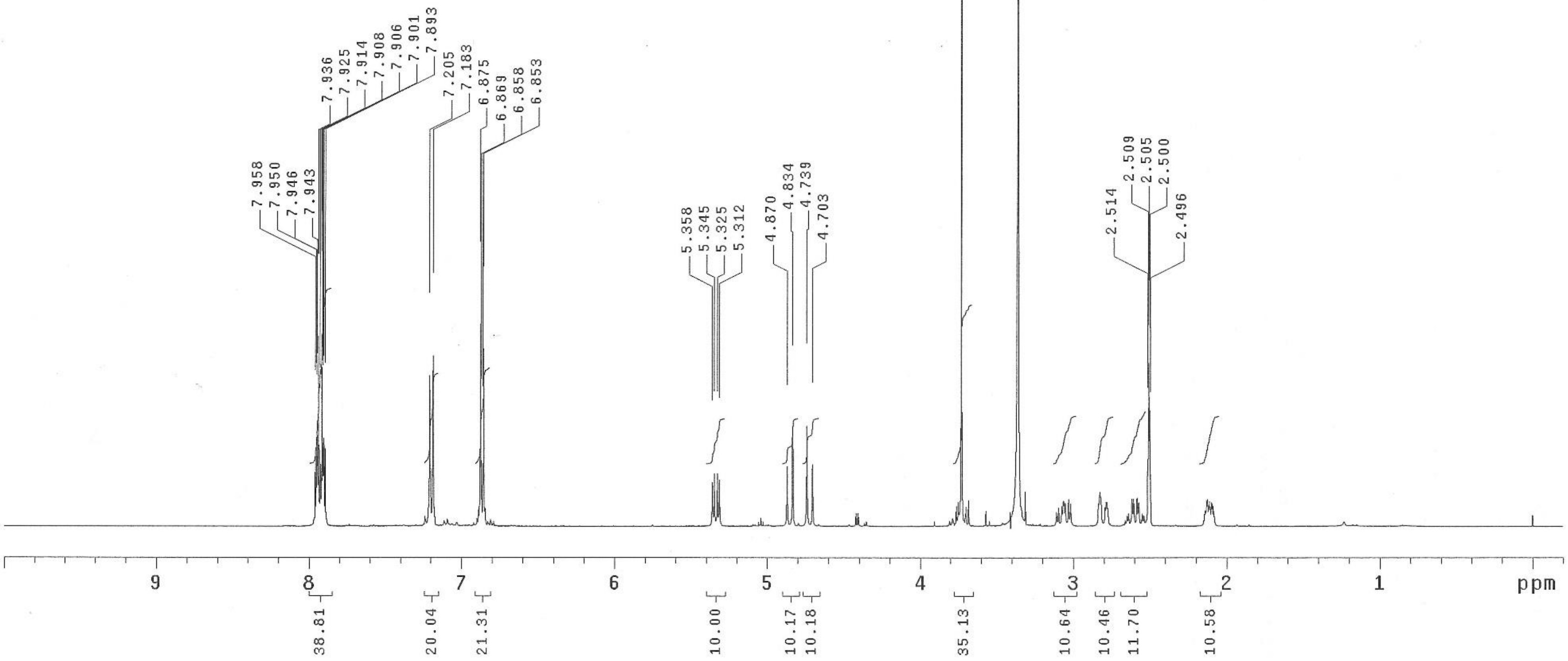

TKW-5659i

Pulse Sequence: s2pul

UNITYplus-400 "unity400"
Date: Aug 17 2016
Solvent: DMSO
Ambient temperature
Total 3200 repetitions

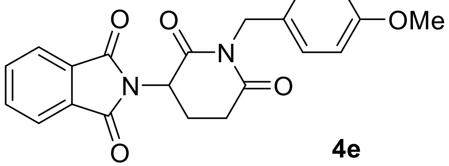


**4e**

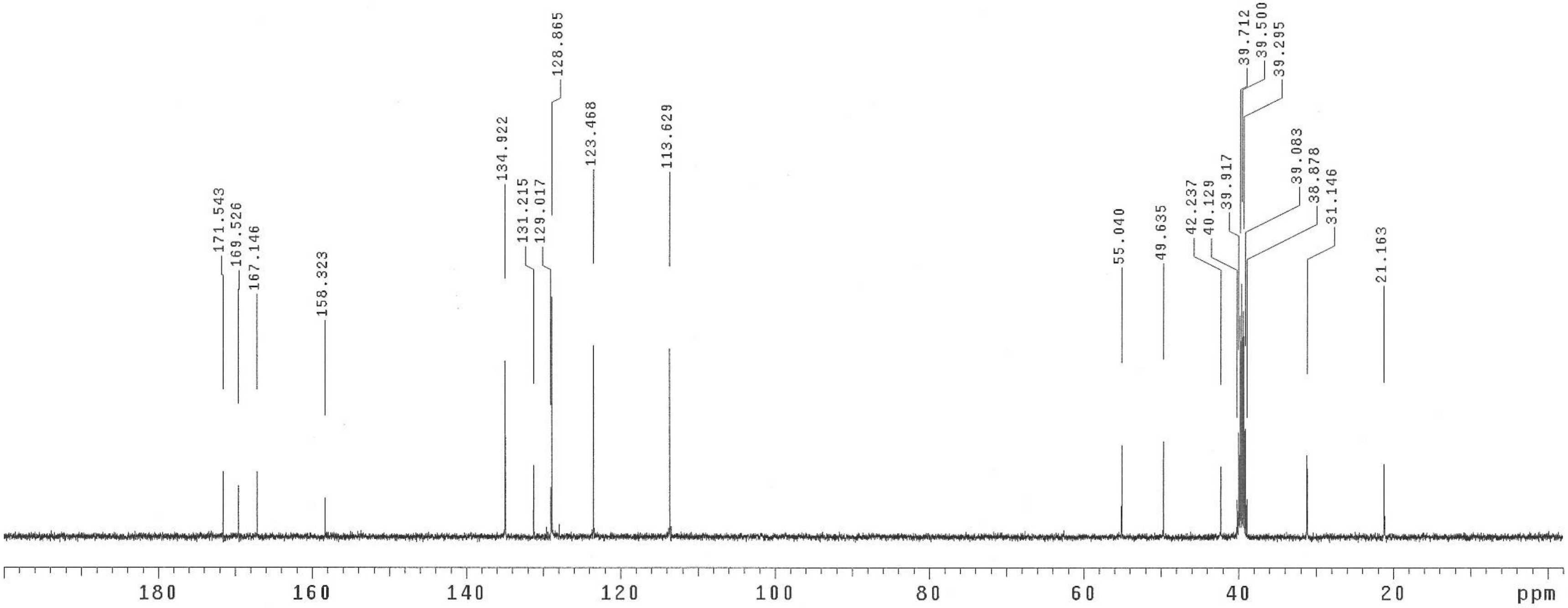

TKW-56621

Pulse Sequence: s2pul
UNITYplus-400 "unity400"
Date: Jun 2 2016
Solvent: DMSO
Ambient temperature
Total 64 repetitions

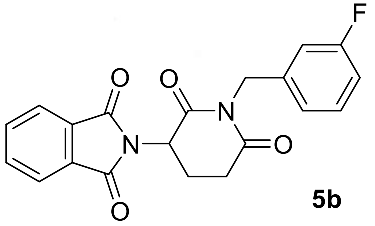

5b

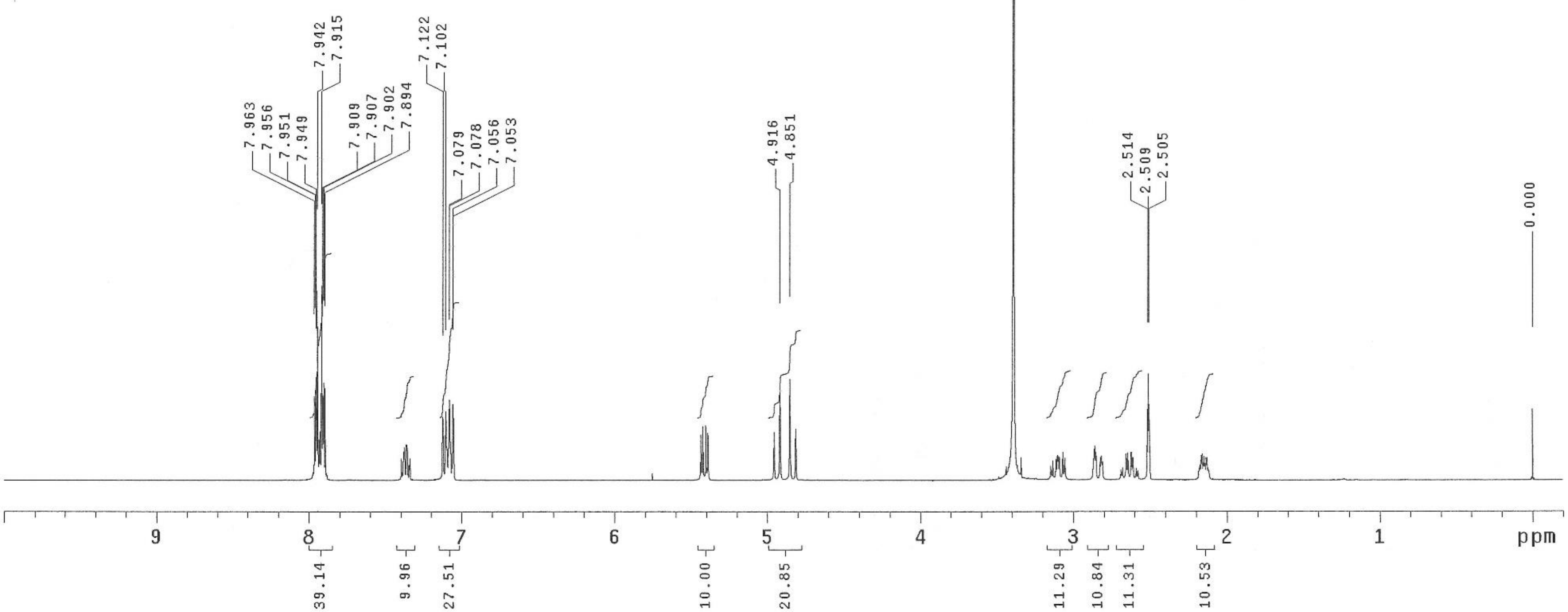

TKW-56621

Pulse Sequence: s2pul
UNITYplus-400 "unity400"
Date: Jun 2 2016
Solvent: DMSO
Ambient temperature
Total 2016 repetitions

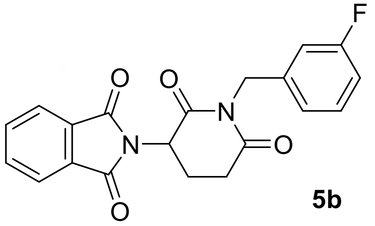

**5b**

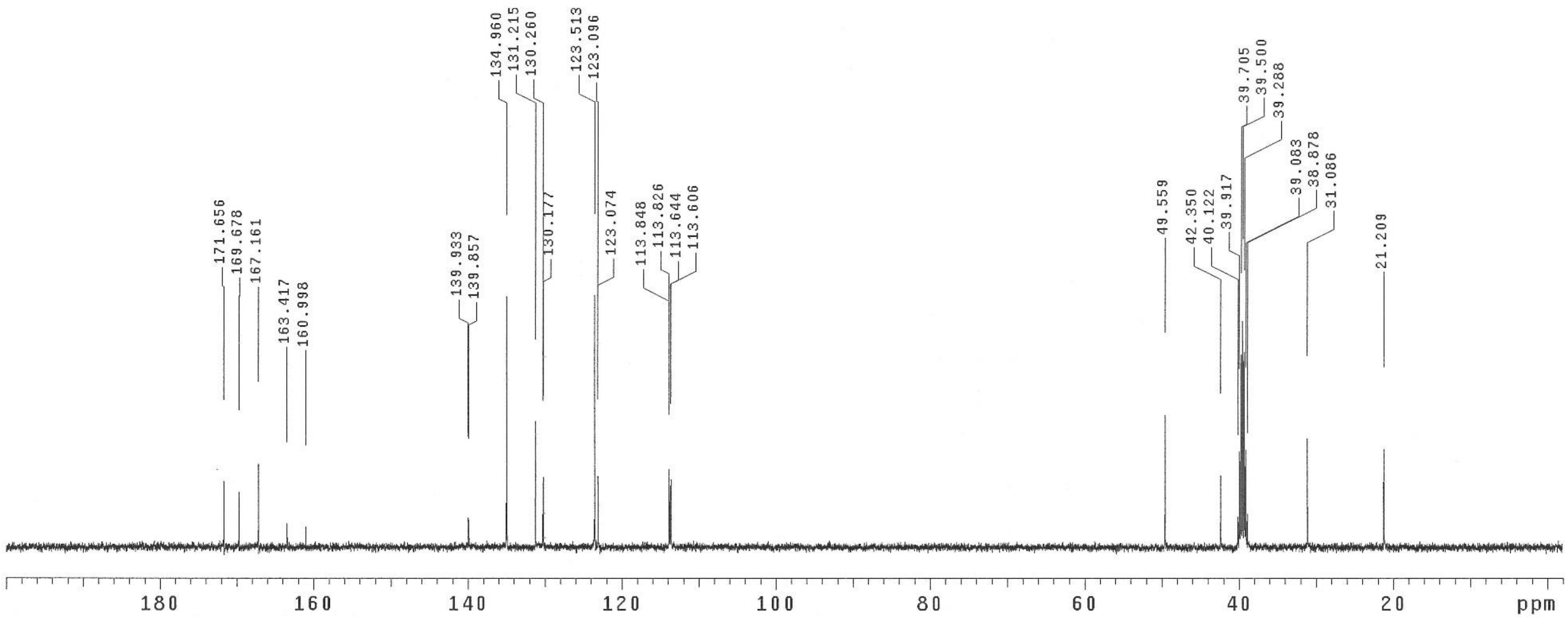

TKW-5664n

Pulse Sequence: s2pul
UNITYplus-400 "unity400"
Date: Jun 3 2016
Solvent: DMSO
Ambient temperature
Total 64 repetitions

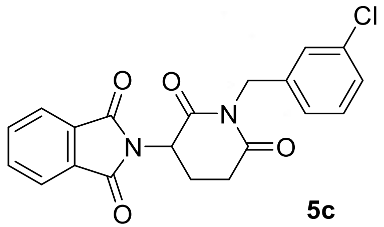

**5c**

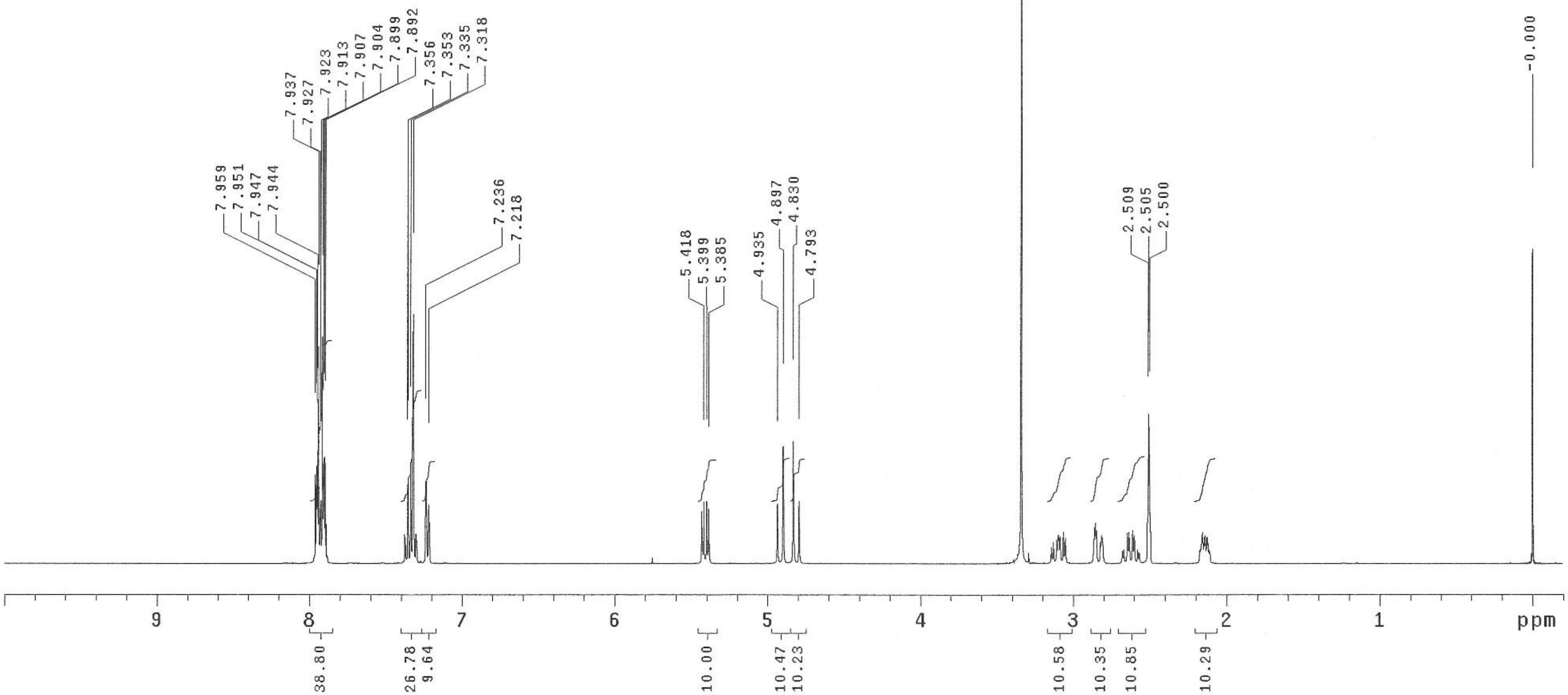

TKW-5664n

Pulse Sequence: s2pul
UNITYplus-400 "unity400"
Date: Jun 3 2016
Solvent: DMSO
Ambient temperature
Total 2272 repetitions

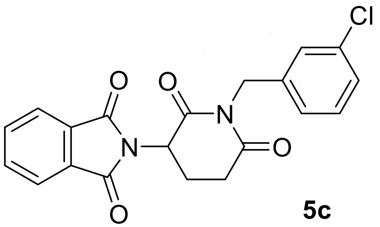

5c

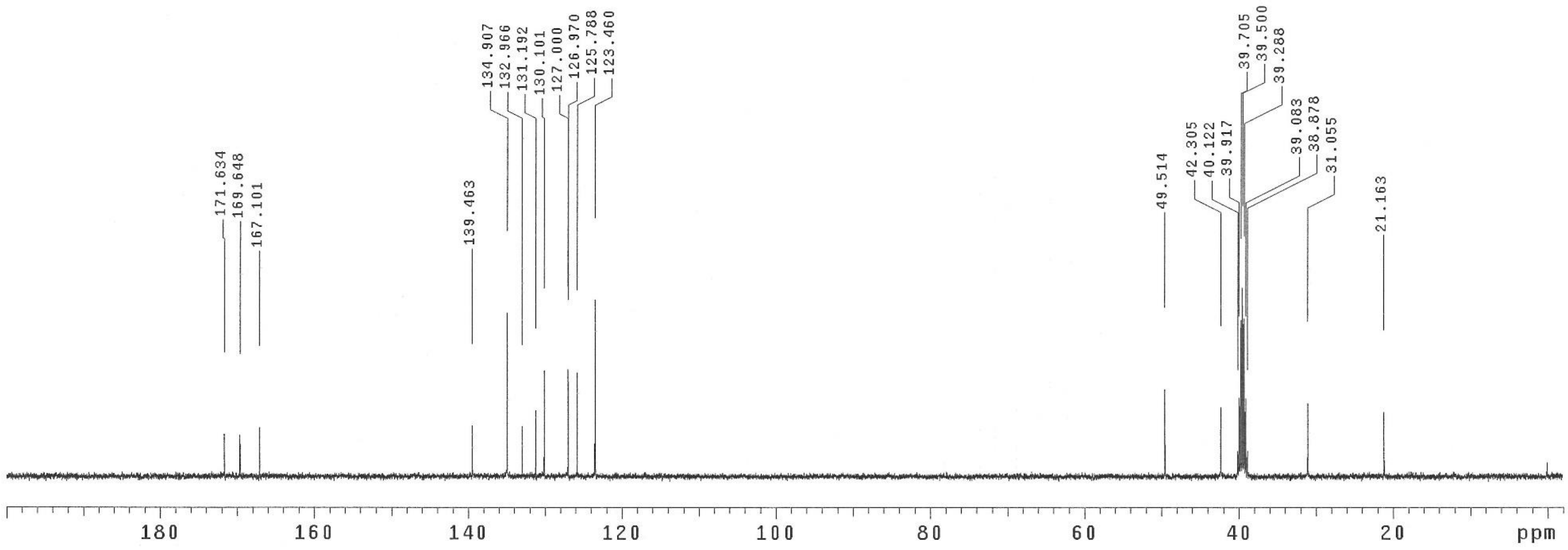

TKW-5663m

Pulse Sequence: s2pul
UNITYplus-400 "unity400"
Date: Jun 3 2016
Solvent: DMSO
Ambient temperature
Total 64 repetitions

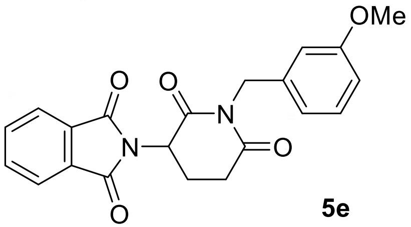


5e

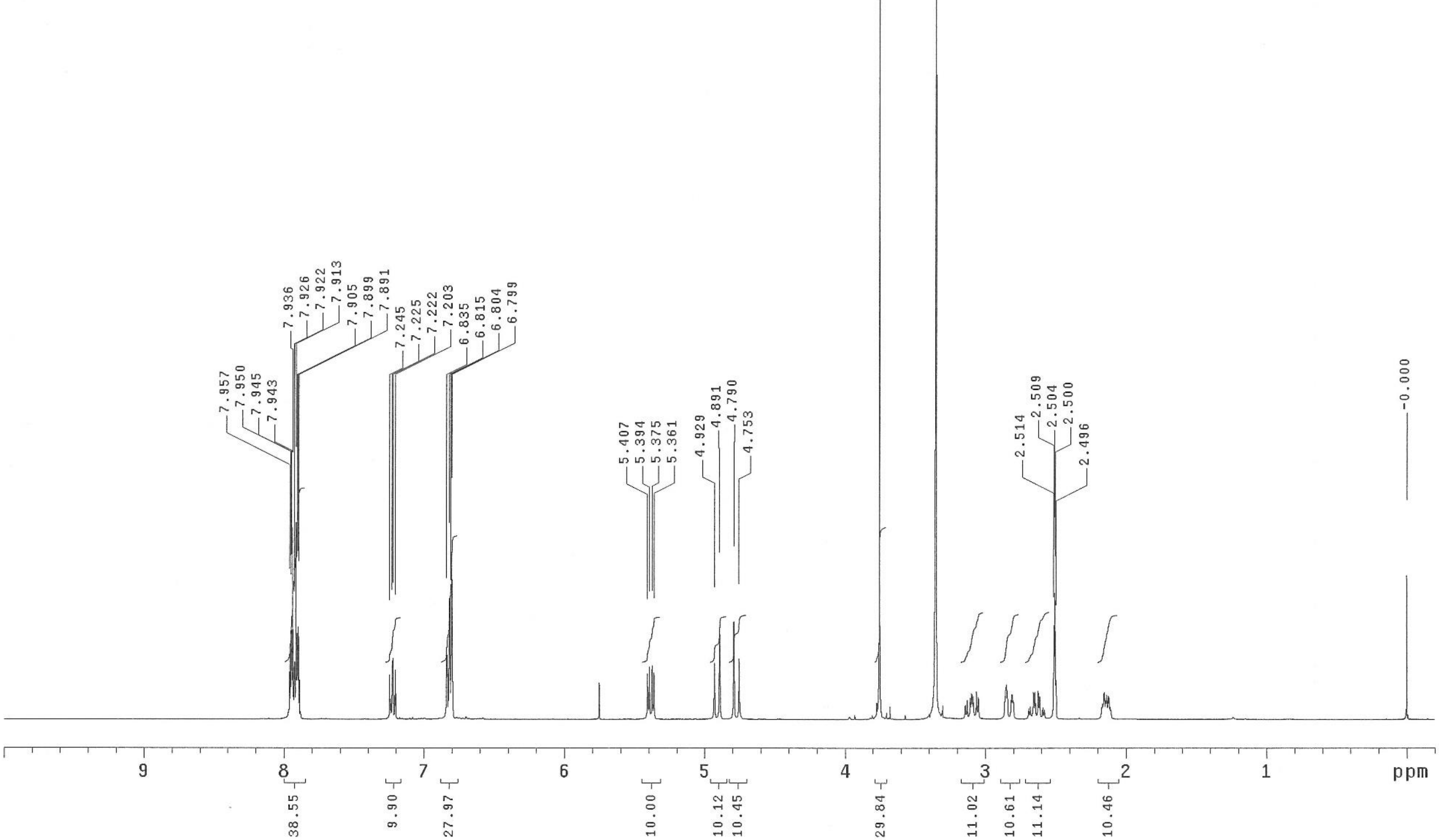

TKW-5663m

Pulse Sequence: s2pul
UNITYplus-400 "unity400"
Date: Jun 3 2016
Solvent: DMSO
Ambient temperature
Total 4096 repetitions

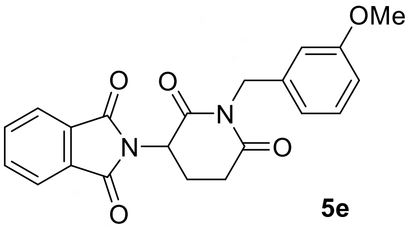


**5e**

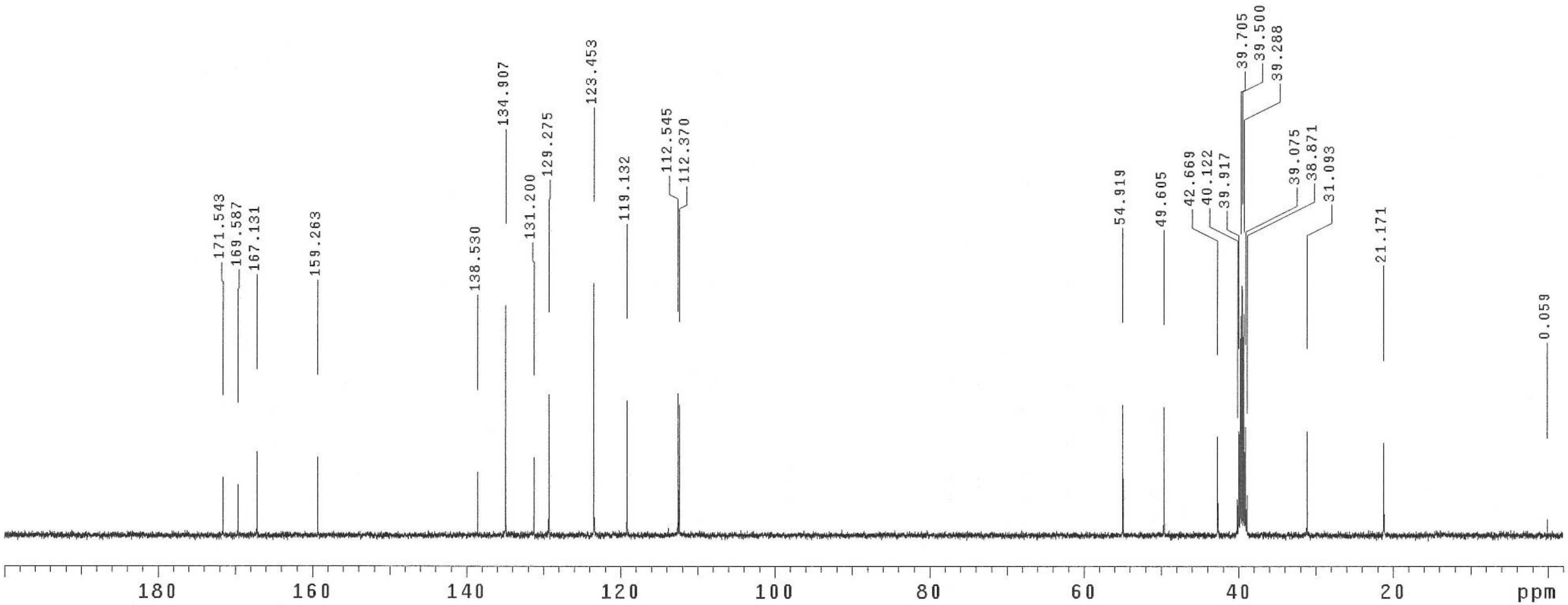

TKW-5665o

Pulse Sequence: s2pul
Mercury-400BB "MerPlus400"
Date: Jun 24 2016
Solvent: dmso
Ambient temperature
Total 64 repetitions

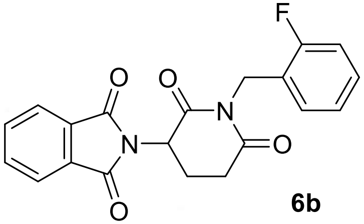

**6b**

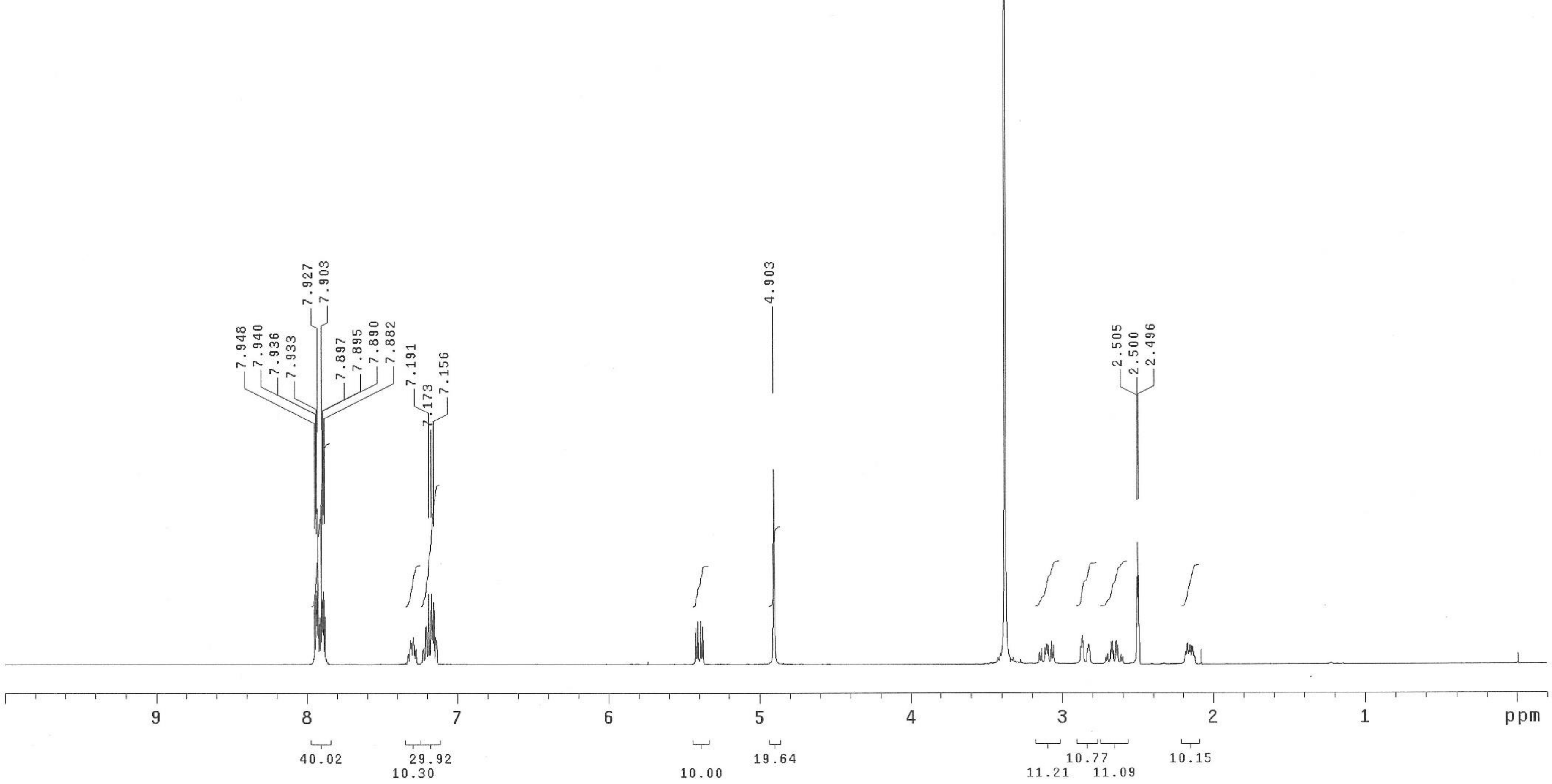

TKW-5665o

Pulse Sequence: s2pul
Mercury-400BB "MerPlus400"
Date: Jun 24 2016
Solvent: dmso
Ambient temperature
Total 64000 repetitions

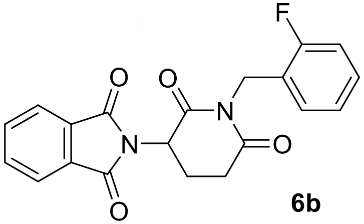


6b

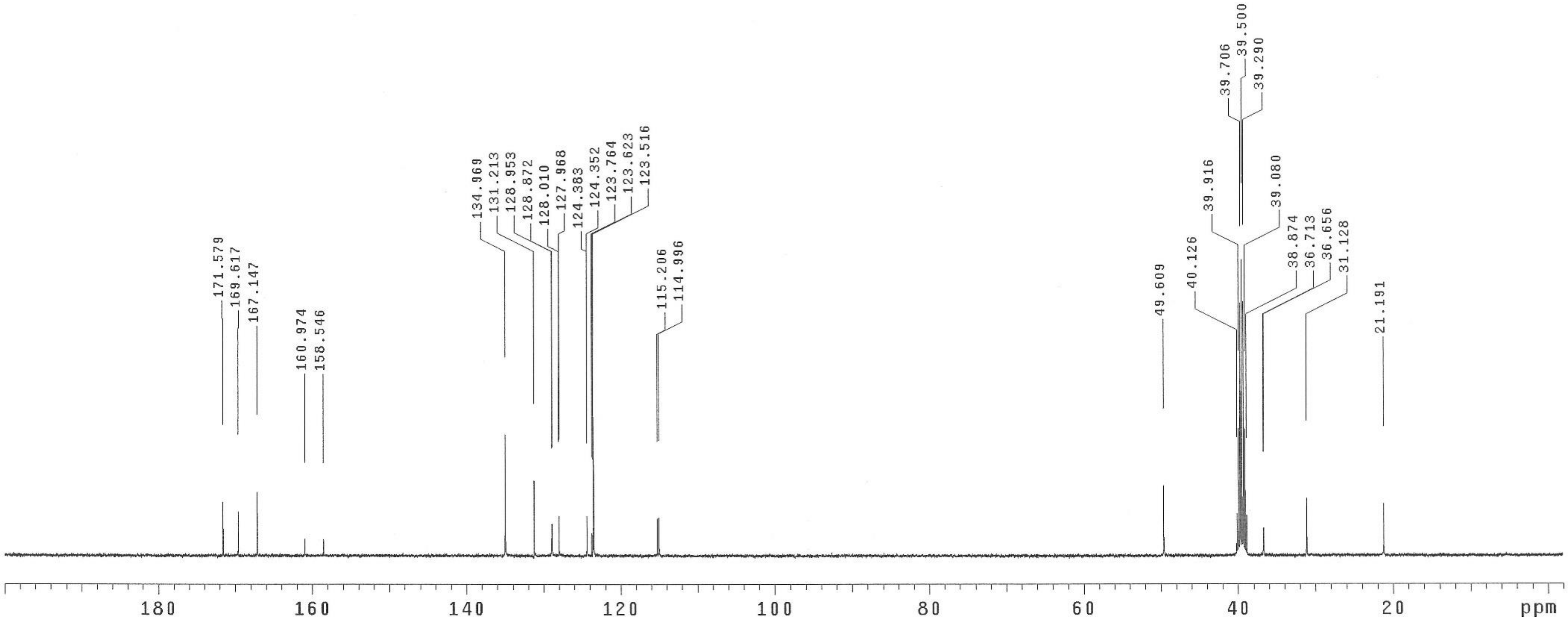

TKW-5682f

Pulse Sequence: s2pul
UNITYplus-400 "unity400"
Date: Nov 11 2016
Solvent: DMSO
Ambient temperature
Total 80 repetitions

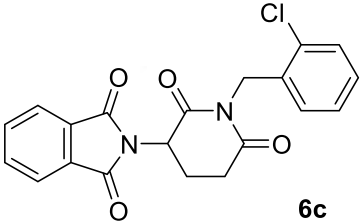


**6c**

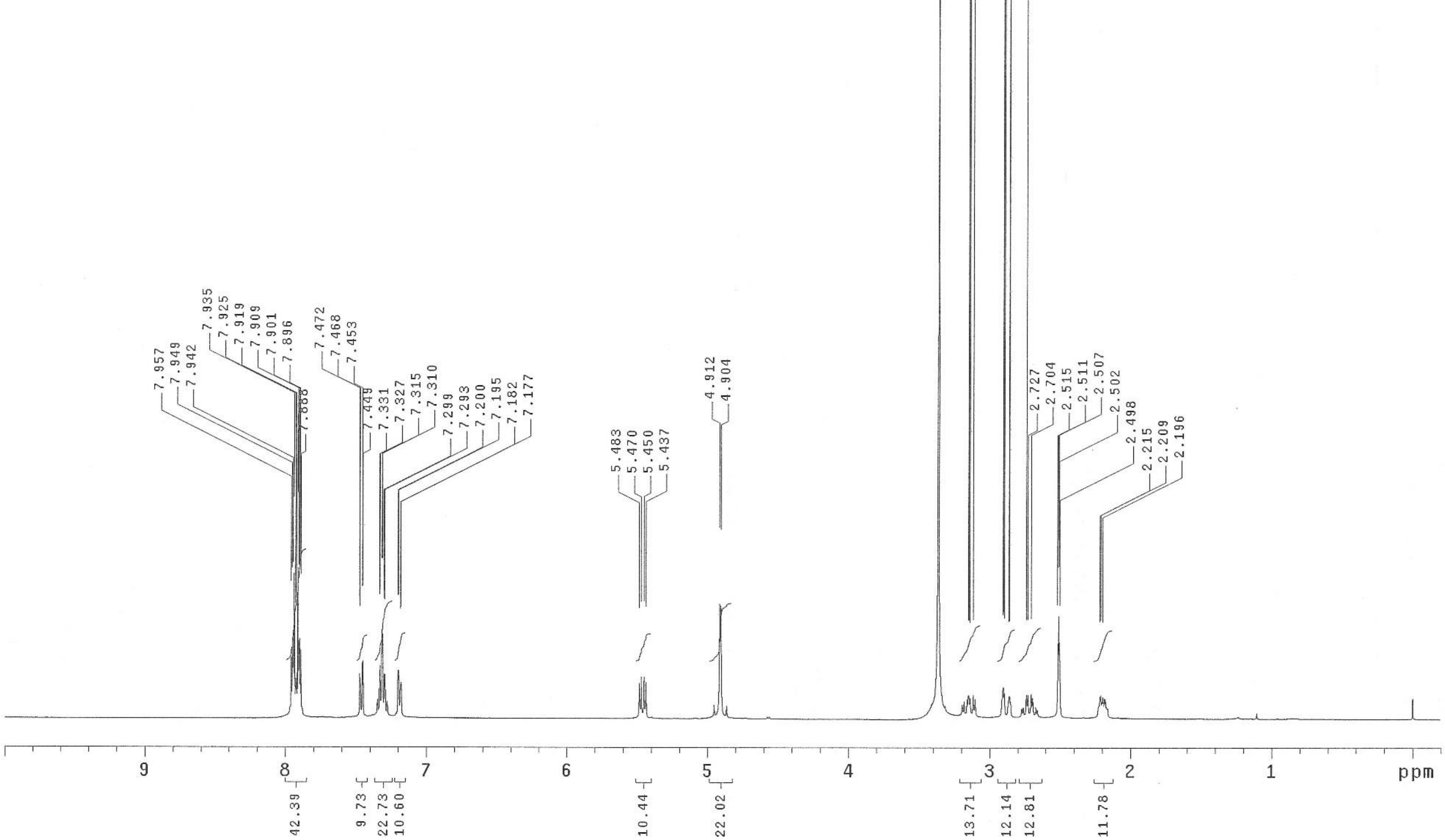

TKW-5682f

Pulse Sequence: s2pul
UNITYplus-400 "unity400"
Date: Nov 11 2016
Solvent: DMSO
Ambient temperature
Total 16000 repetitions

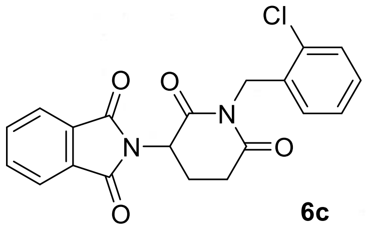

**6c**

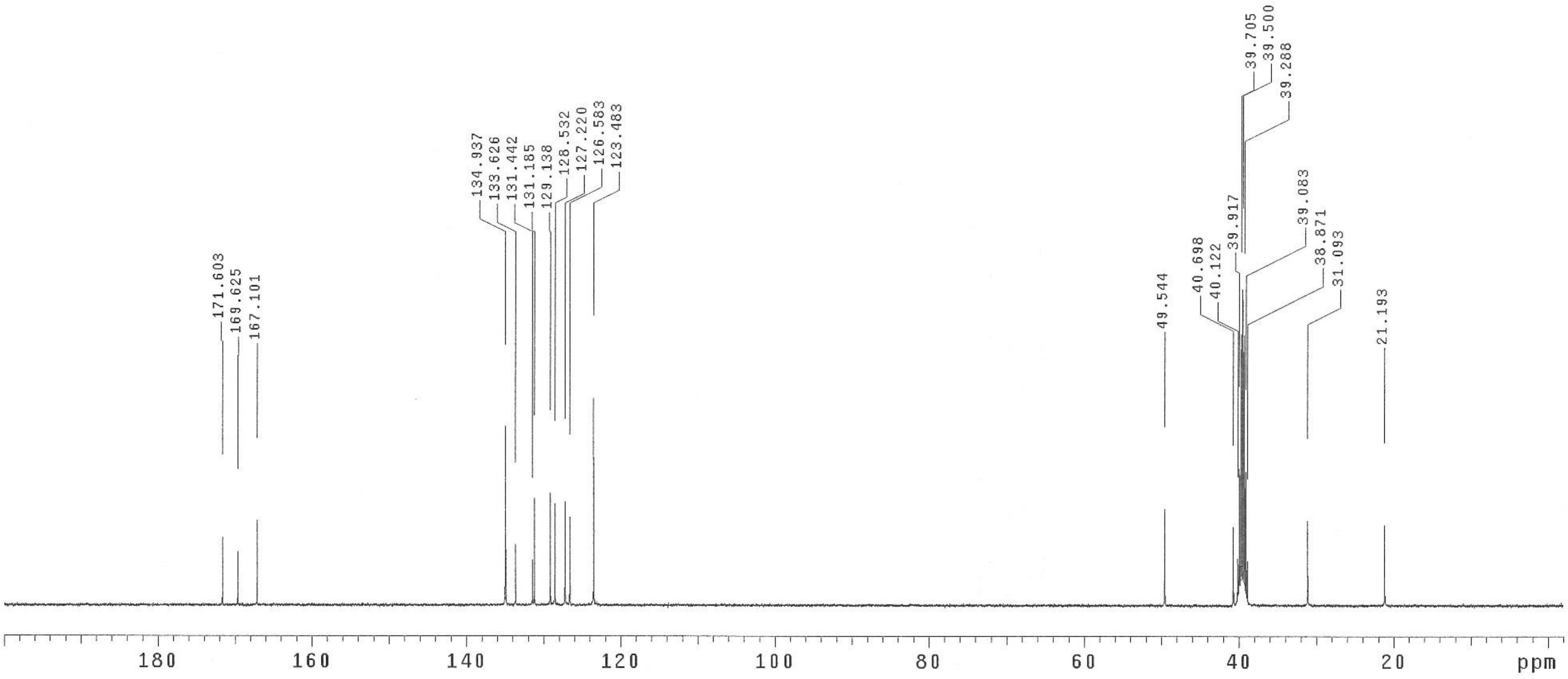

TKW-5916p

Pulse Sequence: s2pul
UNITYplus-400 "unity400"
Date: Jan 26 2018
Solvent: DMSO
Ambient temperature
Total 48 repetitions

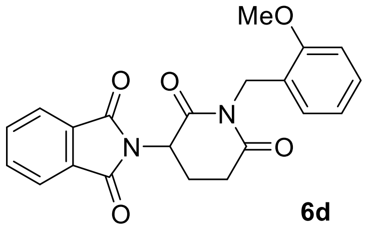


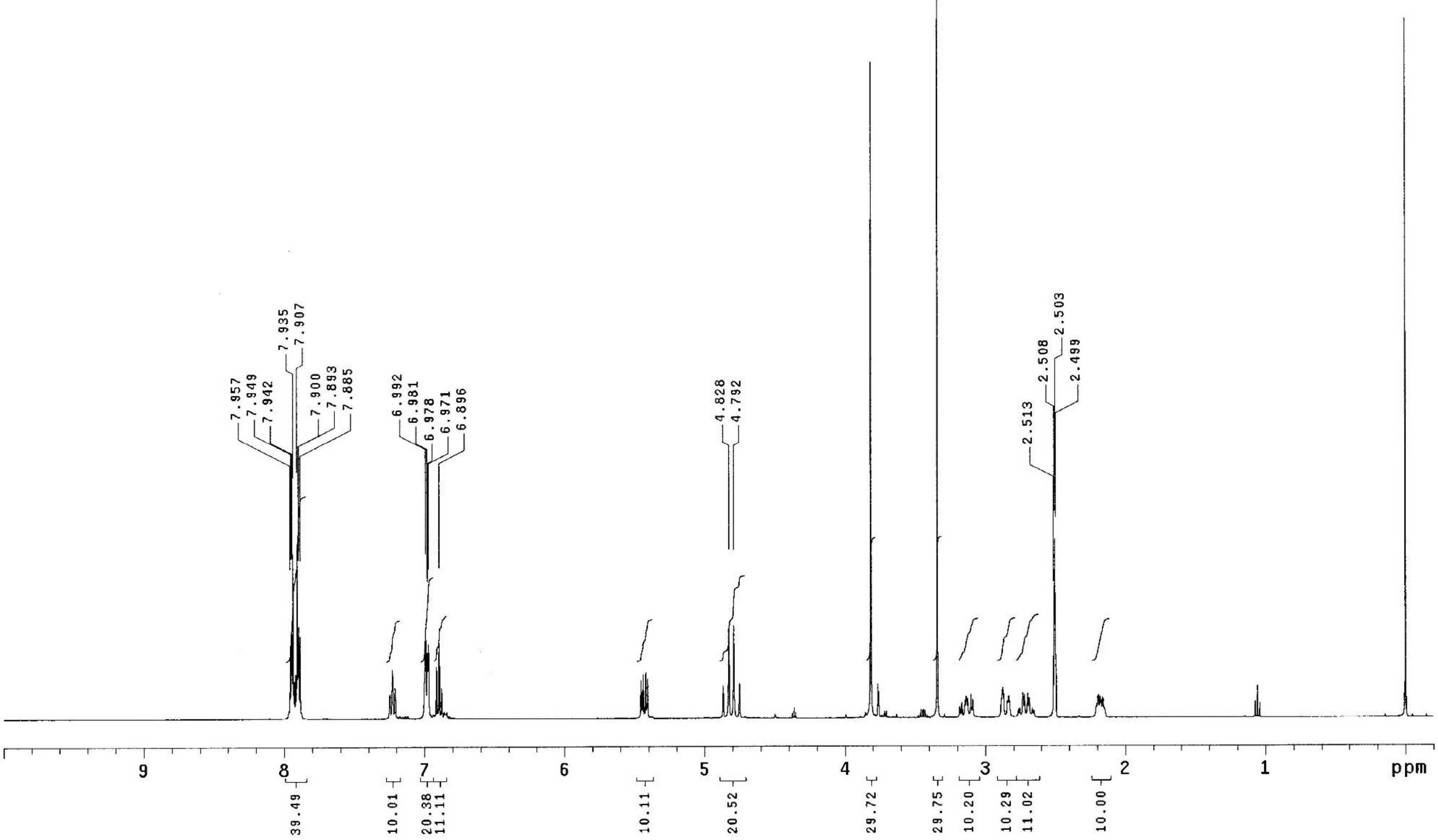

TKW-5916p

Pulse Sequence: s2pul
UNITYplus-400 "unity400"
Date: Jan 26 2018
Solvent: DMSO
Ambient temperature
Total 16000 repetitions

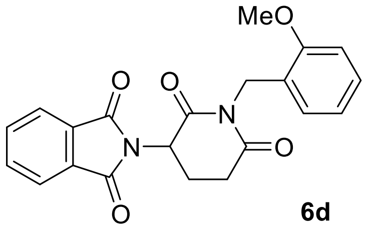


6d

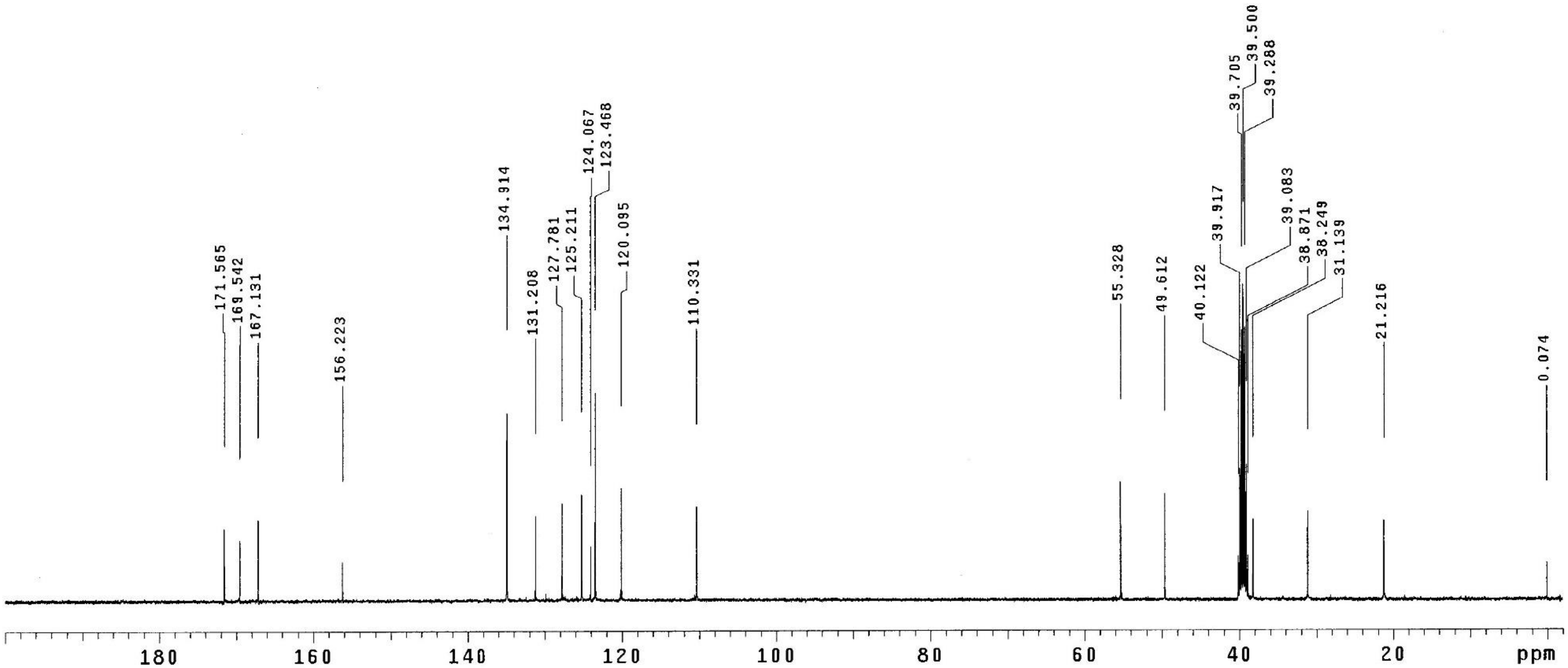

Supplement: Supplementary file 1 [file ijms-19-03061-s001.pdf]
